# Mortality Risk Prediction Models for People With Kidney Failure: A Systematic Review

**DOI:** 10.1001/jamanetworkopen.2024.53190

**Published:** 2025-01-03

**Authors:** Faisal Jarrar, Meghann Pasternak, Tyrone G. Harrison, Matthew T. James, Robert R. Quinn, Ngan N. Lam, Maoliosa Donald, Meghan Elliott, Diane L. Lorenzetti, Giovanni Strippoli, Ping Liu, Simon Sawhney, Thomas Alexander Gerds, Pietro Ravani

**Affiliations:** 1 Department of Medicine, Cumming School of Medicine, University of Calgary, Calgary, Alberta, Canada; 2 Libraries and Cultural Resources, University of Calgary, Calgary, Alberta, Canada; 3 Department of Precision and Regenerative Medicine and Jonian Area, University of Bari, Bari, Italy; 4 School of Public Health, University of Sydney, Sydney, New South Wales, Australia; 5 Aberdeen Centre for Health Data Science, University of Aberdeen, Aberdeen, Scotland, United Kingdom; 6 Department of Public Health, University of Copenhagen, Copenhagen, Denmark

## Abstract

**Importance:**

People with kidney failure have a high risk of death and poor quality of life. Mortality risk prediction models may help them decide which form of treatment they prefer.

**Objective:**

To systematically review the quality of existing mortality prediction models for people with kidney failure and assess whether they can be applied in clinical practice.

**Evidence Review:**

MEDLINE, Embase, and the Cochrane Library were searched for studies published between January 1, 2004, and September 30, 2024. Studies were included if they created or evaluated mortality prediction models for people who developed kidney failure, whether treated or not treated with kidney replacement with hemodialysis or peritoneal dialysis. Studies including exclusively kidney transplant recipients were excluded. Two reviewers independently extracted data and graded each study at low, high, or unclear risk of bias and applicability using recommended checklists and tools. Reviewers used the Prediction Model Risk of Bias Assessment Tool and followed prespecified questions about study design, prediction framework, modeling algorithm, performance evaluation, and model deployment. Analyses were completed between January and October 2024.

**Findings:**

A total of 7184 unique abstracts were screened for eligibility. Of these, 77 were selected for full-text review, and 50 studies that created all-cause mortality prediction models were included, with 2 963 157 total participants, who had a median (range) age of 64 (52-81) years. Studies had a median (range) proportion of women of 42% (2%-54%). Included studies were at high risk of bias due to inadequate selection of study population (27 studies [54%]), shortcomings in methods of measurement of predictors (15 [30%]) and outcome (12 [24%]), and flaws in the analysis strategy (50 [100%]). Concerns for applicability were also high, as study participants (31 [62%]), predictors (17 [34%]), and outcome (5 [10%]) did not fit the intended target clinical setting. One study (2%) reported decision curve analysis, and 15 (30%) included a tool to enhance model usability.

**Conclusions and Relevance:**

According to this systematic review of 50 studies, published mortality prediction models were at high risk of bias and had applicability concerns for clinical practice. New mortality prediction models are needed to inform treatment decisions in people with kidney failure.

## Key Points

**Question:**

What are the quality and clinical applicability of existing mortality prediction models for people with kidney failure?

**Findings:**

This systematic review identified and evaluated 50 studies with more than 2.9 million participants reporting mortality prediction models for people with kidney failure. Models were found to be at high risk of bias and to have applicability concerns for clinical practice, and none demonstrated promise in terms of clinical usability or incorporation into guidelines.

**Meaning:**

These findings suggest that new mortality prediction models are needed to inform treatment decisions in people with kidney failure.

## Teaser

This systematic review evaluates the quality of mortality prediction models for people with kidney failure and assesses their usefulness in clinical practice.

## Introduction

Risk prediction models are increasingly endorsed to help patients understand their treatment preferences and promote personalized care.^1,2^ These models are of particular value for people with kidney failure.^3^ People with kidney failure have higher morbidity and mortality than those with nonmetastatic cancer^4,5^ and often face difficult treatment decisions.^6^ Those who cannot receive a kidney transplant need to consider the trade-off between starting or continuing dialysis therapy (peritoneal dialysis or hemodialysis) and choosing conservative management without dialysis. Personalized risk predictions can inform the management of kidney failure for an individual and support decisions that best reflect their unique goals, preferences, and values.^5,6,7^ Yet, current guidelines do not include any recommendation to consult a mortality prediction model.^8^

One possible barrier to sharing mortality information in clinical practice may be a lack of mortality risk prediction models suitably designed to assist with decision-making.^9,10,11^ Alternatively, promising models may exist that provide potentially relevant and useful information but need further evaluation to determine their clinical applicability. Incorporating a risk prediction model into clinical practice requires evidence that a model is relevant to its intended use, outperforms alternative strategies when challenged with data that it has not been trained on, could feasibly be used, and would help make better informed decisions. For example, consider a model that predicts 1-year mortality risk for people newly diagnosed with kidney failure. Such a model could be used to decide whether a person wants to receive dialysis. The model would need to be designed, created, and evaluated with data that represent the target population of model users—in this case, people newly diagnosed with kidney failure not receiving dialysis.

This systematic review assessed the quality and applicability of mortality prediction models for people with kidney failure. We evaluated whether any of them could be implemented or be adapted for implementation or whether new models are required. We followed critical appraisal guidelines^12,13^ and used recommended tools for assessment of their risk of bias and applicability to the intended population and settings.^14^

## Methods

### Protocol

The protocol of this systematic review (eMethods and eAppendix in Supplement 1) was registered with PROSPERO (CRD42023486220). This systematic review was conducted according to the guidelines set out in the Checklist for Critical Appraisal and Data Extraction for Systematic Reviews of Prediction Modeling Studies (CHARMS)^12^ and the Prediction Model Risk of Bias Assessment Tool (PROBAST)^14^ and was reported according to the Transparent Reporting of Multivariable Prediction Models for Individual Prognosis or Diagnosis reporting guideline for Systematic Reviews of Multivariable Prediction Models (TRIPOD-SRMA).^15^

### Data Sources and Searches

An information specialist and medical librarian (D.L.L.) searched Ovid MEDLINE, Ovid Embase, and the Cochrane Library from 2004, when the Kidney Disease: Improving Global Outcomes was originally established to develop and implement guidelines for the care of people with kidney disease,^16^ to September 30, 2024. Searches combined terms from 3 concepts: (1) chronic kidney failure (eg, *CKD*, *renal insufficiency*); (2) mortality (eg, *mortality*, *death*), and (3) prediction modeling (eg, *calibration*, *measures of discrimination*) and recommended filters for prediction models. Terms were searched as keywords and subject headings (eg, MEDLINE MeSH).^17^ No language restrictions were applied to the search strategy. A complete description of the search strategy is provided in eTable 1 in Supplement 1. The reference lists of all included articles were also searched for any additional, relevant articles.

### Study Selection

This review included studies that created mortality prediction models (all-cause mortality or mortality from specific causes) for people with kidney failure treated with long-term dialysis (ie, hemodialysis or peritoneal dialysis) or people with sustained estimated glomerular filtration rate (eGFR) below 15 mL/min/1.73 m^2^ with a prediction horizon of at least 3 months.^8^ We also considered model evaluation studies to search for the study where the model was created. We excluded studies that were not restricted to people with kidney failure, studies that exclusively included kidney transplant recipients, studies that were limited to patients with acute kidney injury in hospital, and studies that reported associations rather than predictions. We also excluded letters, editorials, narrative reviews, commentaries, and case reports, but their reference lists were used to identify potential primary studies.

### Data Extraction and Quality Assessment

Two reviewers (F.J. and M.P.) independently screened all abstracts of studies in duplicate based on titles and abstracts, and then reviewed full texts to determine eligibility based on inclusion and exclusion criteria. Reviewers followed the CHARMS^12^ and PROBAST^14^ recommendations to extract elements of the prediction framework and analysis strategies as explained later. Reviewers also considered the extent to which each study adhered to the TRIPOD+AI reporting standard for all prediction models, irrespective of whether regression or machine learning methods were used.^13^ Discrepancies between the 2 reviewers in study selection for inclusion and data extraction were resolved by discussing with 2 arbitrators (P.R. and P.L.). Analyses were completed between January and October 2024.

### Elements for Critical Appraisal

Reviewers used the CHARMS checklist (eTable 2 in Supplement 1)^12^ and the PROBAST signaling questions (eTable 3 in Supplement 1)^14^ on the domains of participants, predictors, outcome, and analysis. PROBAST provides a structured approach to rate the risk of bias and applicability concerns as low, high, or unclear.

A medical risk prediction model is developed in a suitable framework that defines the target population, the prediction time origin (when the model is applied to the patient data), the prediction time horizon, the predictors, the outcome, and competing events (if any). The framework implicitly defines who can use the model as well as how and when it can be used.^18^ Risk of bias and applicability concerns are high if study participants, predictors, and outcome do not represent the settings in which the model will be used.

For the risk of bias, PROBAST also includes signaling questions for statistical analysis (analysis domain). Here the focus is on data quality, the statistical methods used to analyze the data, the modeling algorithm that produces the prediction model, and the methods used to evaluate the prediction performance, utility, and usability.

Reviewers assessed the modeling algorithms that the primary studies used to make the prediction model (eMethods in Supplement 1). Reviewers considered modeling algorithms inappropriate if they based the selection of the predictors on univariate analyses and then used expert-driven model building and goodness-of-fit testing to obtain the prediction model.^14,17^ Reviewers also considered backward variable selection as inappropriate unless the modeling algorithm was evaluated using cross-validation in which all steps of the modeling algorithm, including the backward selection, were repeated in training sets and evaluated in independent test sets.^17,19^ Similar considerations apply to the selection of hyperparameters or tuning of machine learning algorithms.^18^

Reviewers assessed whether the primary study used a single split of the data or repeated splits of the data for cross-validation (evaluation using the learning data or internal testing). A single random split is not recommended because the results will typically depend on the random seed (Monte-Carlo error), it is prone to manipulation, and it conceals part of the learning data.^18^ Finally, reviewers assessed whether the final model was evaluated using temporally or geographically distinct data (ie, external testing; eMethods in Supplement 1).

Reviewers extracted the criteria for model evaluation and comparison of rival models as reported by the authors (eMethods in Supplement 1), including calibration plots and the time-dependent area under the receiver operating characteristic curve (AUC), a measure of discrimination, and the time-dependent Brier score or prediction error, a measure of both calibration and discrimination.^18^ Reviewers reported whether the primary study used improper performance measures, including the C index (Harrell concordance index)^20^ and measures of reclassification.^21,22^ These measures are not proper because they may erroneously show that a misspecified model systematically outperforms the data-generating model.^18^

Reviewers assessed whether study authors applied decision curve analysis^23^ or whether they linked specific clinical decisions with categories defined by predicted risks.^24^ Finally, reviewers considered whether a model was tested in a clinical trial, as this is the ultimate test of model utility.^25^ For usability, reviewers noted whether authors provided a calculator, nomogram, or an alternative tool that would ease clinician access to patient predicted risks. Data were collected in Excel version 16.91 for Mac (Microsoft Corp) tables.

## Results

### Study Characteristics

**Figure 1.**
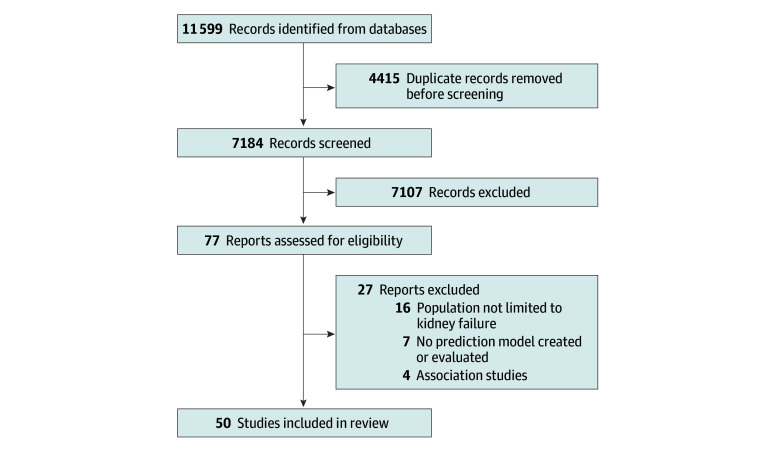
Study Selection

Of the 7184 titles and abstracts screened, 77 studies were selected for full-text review, and 50 articles^26,27,28,29,30,31,32,33,34,35,36,37,38,39,40,41,42,43,44,45,46,47,48,49,50,51,52,53,54,55,56,57,58,59,60,61,62,63,64,65,66,67,68,69,70,71,72,73,74,75^ met the inclusion criteria (Figure 1). These studies reported 50 unique models including a total of 2 963 157 participants (range, 173-1 150 195 participants), with a median (range) age of 64 (52-81) years and median (range) proportion of women of 42% (2%-54%). All models were designed to predict all-cause mortality with time horizons ranging from 3 months to 10 years. Study characteristics are shown in Table 1.

**Table 1.** Characteristics of the 50 Studies Included in the Systematic Review^a^

Source	Design	Enrolment period	Study setting	Study region	Participant No.^b^	Age at entry^c^	Participants, %		
							Women	HD	Incident
Chen et al,^26^ 2014	Retrospective cohort	2005-2010	Multicenter	Taiwan	30 303	64.3 (13.3)	48.4	100	100
Obi et al,^27^ 2018	Retrospective cohort	2007-2015	Multicenter	US	35 878	69.4 (11.1)	2	NA	100
Inaguma et al,^28^ 2019	Prospective cohort	2011-2013	Multicenter	Japan	1520	70 (60.8)	32.4	NA	100
Santos et al,^29^ 2020	Retrospective cohort	2009-2016	Single center	Portugal	421	75.5 (70.8)	46.3	97.6	100
Pladys et al,^30^ 2020	Retrospective cohort	2015	Multicenter	France	9052	68.4 (15.1)	35.6	90.7	100
Hemke et al,^31^ 2013	Prospective cohort	1997-2007	Multicenter	Netherlands	13 868	59.7 (15.1)	38.7	64.5	100
Chen et al,^32^ 2017	Retrospective cohort	2006-2009	Database	US	159 362	NA	46	95.21	100
Chua et al,^33^ 2014	Retrospective cohort	2005-2010	Single center	Singapore	983	60 (13)	47.7	72.4	100
van Diepen et al,^34^ 2014	Prospective cohort	1997-2007	Multicenter	Netherlands	394	65.3 (54.5-72.4)	45	69	100
Floege et al,^35^ 2015	Prospective cohort	2007-2009	Fresenius medical care	Multinational	9722	64.4 (14.7)	40.2	100	100
Dusseaux et al,^36^ 2015	Retrospective cohort	2002-2006	French national registry	France	8955	78 (74-82)	40	NA	100
Doi et al,^37^ 2015	Retrospective cohort	2006-2011	Multicenter	Japan	688	69 (59-77)	33.4	100	0
Thamer et al,^38^ 2015	Retrospective cohort	2009-2010	Multicenter	US	52 796	76.9 (6.5)	46.2	95.8	100
Couchoud et al,^39^ 2015	Retrospective cohort	2005-2012	Multicenter	France	24 348	81.1	39.6	NA	100
Hemke et al,^40^ 2015	Retrospective cohort	1997-2007	Multicenter	Netherlands	1835	59.7 (15.1)	38.7	64.5	100
Patzer et al,^41^ 2016	Retrospective cohort	2005-2011	Multicenter	US	663 860	64.0 (14.9)	44.2	NA	100
Haapio et al,^42^ 2017	Retrospective cohort	2000-2008	Multicenter	Finland	4335	62.3 (21.2)	36.5	76.1	100
Lin et al,^43^ 2019	Retrospective cohort	2000-2011	Multicenter	Taiwan	48 153	75 (69.5-79.0)	54	NA	NA
Akbilgic et al,^44^ 2019	Retrospective cohort	2007-2014	Multicenter	US	27 615	68.7 (11.2)	1.9	94	100
Cho et al,^45^ 2017	Retrospective cohort	2005-2008	Multicenter	South Korea	7606	54.5 (13.8)	44	0	100
Wick et al,^46^ 2017	Retrospective cohort	2003-2012	Multicenter	Canada	2199	75.2 (6.5)	39.2	85.5	100
Ivory et al,^47^ 2017	Retrospective cohort	2000-2009	Multicenter	Australia, UK, New Zealand	23 658	NA	40	NA	100
Geddes et al,^48^ 2006	Combined data^d^	1997-2002	Multicenter	Multinational	2310	63.4 (13.5)	42.3	NA	100
Mauri et al,^49^ 2008	Prospective cohort	1997-2003	Multicenter	Spain	5738	64.6 (14.4)	37.8	100	100
Couchoud et al,^50^ 2009	Retrospective cohort	2002-2006	Multicenter	France	4991	80.9 (4.1)	39.4	NA	100
Liu et al,^51^ 2010	Retrospective cohort	1999-2005	Multicenter	US	33 077	65 (15)	48.1	NA	100
Jacob et al,^52^ 2010	Prospective cohort	1990-2007	Multicenter	US	242 576	NA	46.68	89.23	100
Marinovich et al,^53^ 2010	Retrospective cohort	2004-2005	Multicenter	Argentina	5360	58.9 (15.5)	43.8	100	100
Quinn et al,^54^ 2011	Retrospective cohort	1998-2005	Multicenter	Canada	16 205	62.81 (15.7)	41.64	75.75	100
Wu et al,^55^ 2022	Retrospective cohort	2006-2015	Multicenter	Taiwan	210 174	NA	42.6	NA	100
Noh et al,^56^ 2020	Retrospective cohort	2008-2014	Multicenter	Korea	1730	52.7 (12.6)	42.7	0	NA
Siddiqa et al,^57^ 2021	Retrospective cohort	2013-2017	Multicenter	Pakistan	758	NA	NA	100	NA
McAdams-DeMarco et al,^58^ 2018	Retrospective cohort	2009-2016	Multicenter	US	1975	53.7 (13.5)	40.5	67.7	NA
Gao et al,^59^ 2022	Retrospective cohort	2011-2019	Multicenter	China	200	52.26 (13.3)	45	60.5	NA
Chaudhuri et al,^60^ 2023	Retrospective cohort	2000-2019	Multicenter	Global	76 113	61.7	42.6	100	NA
Thijssen et al,^61^ 2012	Retrospective cohort	2000-2009	Multicenter	US	4512	61.3 (15.5)	43.4	100	100
Wagner et al,^62^ 2011	Retrospective cohort	2002-2004	Multicenter	UK	3631	64 (49.73)	37.8	70.1	100
Zhu et al,^63^ 2021	Retrospective cohort	2010-2016	Single center	China	173	58	30	100	100
Cohen et al,^64^ 2010	Prospective cohort	2006-2008	Multicenter	US	512	61 (17)	44	100	NA
Siga et al,^65^ 2020	Prospective cohort	2010-2014	Multicenter	France	4915	NA	NA	100	100
Jung et al,^66^ 2018	Prospective cohort	2008-2011	Multicenter	Korea	3309	61.7 (13.6)	41.2	71.2	NA
Wang et al,^67^ 2021	Retrospective cohort	2007-2016	Multicenter	China	1200	55 (NA)	36.5	100	NA
Tapak et al,^68^ 2020	Retrospective cohort	2007-2017	Multicenter	Iran	785	NA	45.1	100	100
Holme et al,^69^ 2012	Prospective cohort (secondary analysis)	2003-2004	Multicenter	International	1868	64 (8.6)	38	100	0
Rankin et al,^70^ 2022	Retrospective cohort	2008-2017	Multicenter	US	1 150 195	63 (15)	42	NA	100
Fernandez Lucas et al,^71^ 2007	Retrospective cohort	2000-2004	No information	Spain	304	64 (16-84)	37	80	100
Goldstein et al,^72^ 2024	Retrospective cohort	2003-2013	Single center	US	42 351	63.4 (52.5-73.6)	43	100	100
Noppakun et al,^73^ 2024	Retrospective cohort	2005-2016	Thai registry	Thailand	17 354	76.9 (5.1)	53.5	100	100
Yang et al,^74^ 2023	Retrospective cohort	2007-2020	Single center	China	551	61 (51-70)	47.2	100	100
Okada et al,^75^ 2024	Retrospective cohort	2006-2007	Japanese registry	Japan	2739	80 (77,83)	42.7	100	100

Abbreviations: HD, hemodialysis; NA, not available.

^a^ No study included people with kidney failure not treated with kidney replacement. All studies were written in English language.

^b^ Participant number refers to the number of incident and/or prevalent patients receiving dialysis. As opposed to incident patients for whom the study entry date corresponded to the dialysis start date, prevalent patients had been receiving dialysis for a variable amount of time when they entered the study. One study (Patzer et al,^41^ 2016) included transplant recipients (whose data were not included in this review).

^c^ Age in years summarized as mean (SD) or median (range).

^d^ Design combined retrospective and prospective data.

### Adherence to Recommended Reporting Guidelines

None of the studies met all criteria of the TRIPOD+AI statement for methods. Sample size and power considerations were not reported in any of the studies, and information on whether there were missing data and how they were handled was not reported in 26 studies (52%).^26,28,29,33,43,45,46,49,51,53,54,57,58,59,60,61,62,63,66,67,68,69,71,72,73,74^ As for the TRIPOD+AI results checklist, none of the studies met all criteria. Criteria for model specification was not met in 28 studies (56%)^26,28,30,32,36,43,44,45,46,50,51,52,53,54,55,56,57,63,64,65,66,67,68,69,71,73,75^ and participants in 34 studies(68%).^26,28,32,33,34,37,39,43,44,45,46,47,49,51,52,53,54,56,57,58,59,60,61,63,64,65,66,67,68,69,71,72,73,74^ None of the studies met all criteria for usability of the model in the context of current care. Data related to TRIPOD+AI recommendations are shown in eTable 4 in Supplement 1.

### Prediction Framework

All studies included people who had already made a treatment choice for kidney failure, ie, dialysis therapy. None included people who had chosen conservative care without dialysis or people who still had to decide their preferred treatment. Most studies (39 [78%])^26,27,28,29,30,31,32,33,34,35,36,38,39,40,41,42,44,45,46,47,48,49,50,51,52,53,54,55,61,62,63,65,68,70,71,72,73,74,75^ included incident patients who entered the study on the dialysis start date. Two studies (4%)^37,69^ included prevalent patients who had been receiving dialysis for a variable time before the enrolment date. The remaining 9 studies (18%)^43,56,57,58,59,60,64,66,67^ did not specify whether patients were prevalent patients already receiving dialysis or incident patients who started dialysis at study entry. Information on the type of dialysis was not reported in 12 studies (24%),^27,28,36,39,41,43,47,48,50,51,55,70^ 18 studies (36%)^26,35,37,49,53,57,60,61,63,64,65,67,68,69,72,73,74,75^ included only patients receiving hemodialysis, 2 studies (4%)^45,56^ only patients receiving peritoneal dialysis patients, and the remaining (18 studies [36%]^29,30,31,32,33,34,38,40,42,44,46,52,54,58,59,62,66,71,76^) a variable proportion of the 2 dialysis modalities. All studies used a prediction framework with a single time origin (cohort entry). In 16 studies (32%),^34,43,51,56,57,58,59,60,62,63,64,65,66,67,68,69^ the prediction time origin was not described, and 3 studies (6%)^51,58,72^ did not specify a prediction time horizon. Sixteen studies (32%)^26,29,51,52,56,58,59,60,61,63,65,66,67,68,69,71^ did not describe the intended use of their model. Four studies (8%)^31,35,40,45^ used predictors measured after the prediction time origin, while 11 studies (22%)^35,43,56,57,59,63,64,65,66,67,68^ did not report when predictors were measured. Prediction framework data are summarized in Table 2 and detailed in eTable 5 in Supplement 1.

**Table 2.** Studies Meeting Criteria for Optimal Prediction Framework Design, Model Training and Testing Strategies, and Measures of Prediction Performance and Usefulness

Criterion	Criterion definition	Studies addressing each item
**Prediction framework**		
Target population	Describes whether the study addressed or specified for whom the prediction model is intended to be used. Study outlines which inclusion and exclusion criteria should be applied when using the model, eg, if making predictions in people starting dialysis (target population), the prediction model should be trained and tested in incident patients.	Kidney replacement: 38 studies (76%)^26,29,30,31,32,33,34,35,37,38,40,42,44,45,46,49,52,53,54,56,57,58,59,60,61,62,63,64,65,66,67,68,69,71,72,73,74,75^; kidney failure not treated with kidney replacement: 0 studies; unclear: 12 studies (24%)^27,28,36,39,41,43,47,48,50,51,55,70^
Target of the analysis	Describes the parameter the study aimed to estimate (eg, individualized risk of death). The analysis target is the mortality risk prediction for an individual.	All clearly defined the event of death; none identified the target of the analysis as the predicted risk for an individual
Time origin for prediction	Reflects when time zero of survival analysis was in the study. Does the time zero in the study reflect the time when predictions are made? Is time zero a common time point in participants’ disease trajectory?	First dialysis: 30 studies (60%)^26,27,28,29,30,32,33,36,37,38,39,41,42,44,46,47,48,49,50,52,53,54,55,61,70,71,72,73,74,75^; after dialysis start: 4 studies (8%)^31,35,40,45^; unclear: 16 studies (32%)^34,43,51,56,57,58,59,60,62,63,64,65,66,67,68,69^
Prediction time horizons	Describes what time horizons were chosen in the study, ie, how far in time from the time origin the prediction is projected (eg, 10-y survival probability). Do the time horizons reflect a range that covers age-dependent patient needs (early for older people and more distant for younger people)?	Not reported: 3 studies (6%)^51,58,72^; reported: 47 studies (94%)^26,27,28,29,30,31,32,33,34,35,36,37,38,39,40,41,42,43,44,45,46,47,48,49,50,52,53,54,55,56,57,59,60,61,62,63,64,65,66,67,68,69,70,71,73,74,75^
Static vs dynamic risk prediction	Describes whether the models are meant to be used once at baseline or repeatedly over time (ie, static vs dynamic). If repeatedly, was immortal time addressed by landmarking analysis?	Static: 50 studies (100%)^26,27,28,29,30,31,32,33,34,35,36,37,38,39,40,41,42,43,44,45,46,47,48,49,50,51,52,53,54,55,56,57,58,59,60,61,62,63,64,65,66,67,68,69,70,71,72,73,74,75^; dynamic: 0 studies
Outcome of interest	Describes the event of interest (eg, all-cause death, disease-specific death). Does the definition and the method to capture death parallel real-life situation?	All-cause death: 50 studies (100%)^26,27,28,29,30,31,32,33,34,35,36,37,38,39,40,41,42,43,44,45,46,47,48,49,50,51,52,53,54,55,56,57,58,59,60,61,62,63,64,65,66,67,68,69,70,71,72,73,74,75^
Competing risks	Describes whether competing risks were considered, if applicable.	Not applicable to all-cause mortality
Intended use	Describes who is going to use the model and how. Did the authors specifically address who the intended model users are?	Described: 34 studies (68%)^27,28,30,31,32,33,34,35,36,37,38,39,40,41,42,43,44,45,46,47,48,49,50,53,54,55,57,62,64,70,72,73,74,75^; unclear: 16 studies (32%)^26,29,51,52,56,58,59,60,61,63,65,66,67,68,69,71^
**Model testing**		
Internal testing	Describes whether internal testing techniques were used by the authors. Only cross-validation is appropriate. Measured by statistics of model performance, such as discrimination and calibration.	Not performed: 16 studies (32%)^33,35,36,38,42,45,47,48,51,53,58,59,63,64,66,71^; performed: 34 studies (68%),^26,27,28,29,30,31,32,34,37,39,40,41,43,44,46,49,50,52,54,55,56,57,60,61,62,65,67,68,69,70,72,73,74,75^ with 21 (62%)^27,30,31,32,39,40,41,49,50,52,56,57,60,61,62,69,70,72,73,74,75^ single split, 7 (21%)^26,43,44,46,55,65,67^ cross-validation, and 6 (18%)^28,29,34,37,54,68^ bootstrapping
External testing	Describes whether the authors tested the performance of the model using data not seen during model training.	Performed: 13 studies (26%),^27,35,36,38,42,45,47,48,51,55,57,64,74^ with 1 study (8%)^47^ using temporally and geographically distinct data; 8 studies (62%)^36,38,42,48,51,55,64,75^ using temporally distinct data only; and 4 studies(31%)^27,35,45,57^ using data from different data sources
**Model performance**		
Performance measures	Common performance measures, including the time-dependent AUC and Brier score (prediction error, a measure of both calibration and discrimination), were considered.	C index: 43 studies (86%)^26,27,29,30,31,32,33,34,39,40,41,42,43,44,45,46,47,49,50,51,53,54,55,56,57,58,59,60,61,62,63,64,65,66,67,68,69,70,73,75^; AUC graph: 22 studies (44%)^28,29,30,34,35,44,45,46,48,53,59,60,61,63,65,66,67,70,71,72,74,75^; Brier score: 2 studies (4%)^47,63^; calibration plot: 25 studies (50%)^26,27,30,31,34,35,36,37,38,40,41,42,46,47,50,54,55,62,63,65,66,70,72,74^
**Clinical usefulness**		
Utility	Do the authors discuss a threshold risk or risk category above which a diagnostic or therapeutic decision is made. Is there a decision curve analysis showing net benefit of using the model?	Met criteria: 1 study (2%)^74^
Usability	There is an easy-to-use risk calculator or nomogram that facilitates risk prediction at the bedside.	Met criteria: 15 studies (30%)^27,29,30,34,35,37,39,41,46,47,55,59,60,63,74^

Abbreviation: AUC, area under the receiver operating characteristic curve.

### Modeling Algorithms and Evaluation of Prediction Performance

Primary studies used traditional and/or machine learning methods for prediction model creation (eTable 6 in Supplement 1), including logistic regression (19 studies [38%]^29,31,32,33,34,36,37,38,39,41,42,46,47,49,50,54,55,61,75^), Cox regression (21 studies [42%])^26,27,28,30,35,40,45,48,51,53,57,58,59,62,63,64,66,69,71,73,74^, and random forests (1 study [2%]^68^) for survival outcomes. Six (12%) used machine learning algorithms for binary outcomes: random forests (1 study [2%]^44^), random forests and neural networks (1 study [2%]^43^), extreme gradient boosting machines (2 studies [4%]^60,7^), logistic regression and Bayesian networks (1 study [2%]^65^), and neural networks (1 study [2%]^67^). Two studies (4%)^56,72^ used regression methods, random forests, extreme gradient boosting, and neural networks for binary and survival outcomes. One study (2%)^52^ used linear regression and neural networks for continuous outcomes (time). None of the studies that used methods for binary outcomes provided information on how censoring was handled. Most predictor variable selection strategies were based on univariate analyses. Of the 21 studies (42%)^26,27,31,33,34,37,38,40,41,42,46,50,52,54,55,59,60,64,69,72,73^ that applied automatic variable selection methods, 7 studies (33%)^27,34,37,40,46,54,73^ accounted for optimism, but none used cross-validation to evaluate the prediction performance of the selected prediction model. Results of internal evaluation of the prediction performance were reported in 34 studies (68%), of which 7 studies (21%)^26,43,44,46,55,65,67^ used *k*-fold cross-validation, 6 studies (18%)^28,29,34,37,54,68^ used a not otherwise explained bootstrapping procedure, and 21 studies (62%)^27,30,31,32,39,40,41,49,50,52,56,57,60,61,62,69,70,72,73,74,75^ used a single random split of the data. Results of an external evaluation of the prediction performance were reported in 13 studies (26%). One of these studies (8%)^47^ tested the model using temporally distinct data and geographically distinct data, 8 (62%)^36,38,42,48,51,55,64,75^ used temporally distinct data, and 4 (31%)^27,35,45,57^ used data from different data sources (eTable 5 in Supplement 1).

### Performance Measures

The most used measures of prediction performance were measures of discrimination ability, including the C index and the time-independent AUC (41 studies [82%]^26,27,28,29,30,31,32,33,34,35,36,37,38,39,40,41,42,45,46,47,48,49,50,51,53,54,55,57,58,59,61,62,63,64,66,68,69,71^). A calibration plot was reported in 24 studies (48%).^26,27,30,31,34,35,36,37,38,40,41,42,46,47,50,54,55,62,63,65,66,70,72,74^ Two studies (4%)^47,63^ reported Brier scores (eTable 6 in Supplement 1).

### Clinical Usefulness

Criteria for usability (publication of a risk calculator or nomogram) were met in 15 studies (30%)^27,29,30,34,35,37,39,41,46,47,55,59,60,63,74^ (eTable 5 in Supplement 1). One study (2%)^74^ reported decision curve analysis.

### Risk of Bias and Applicability Concerns

**Figure 2.**
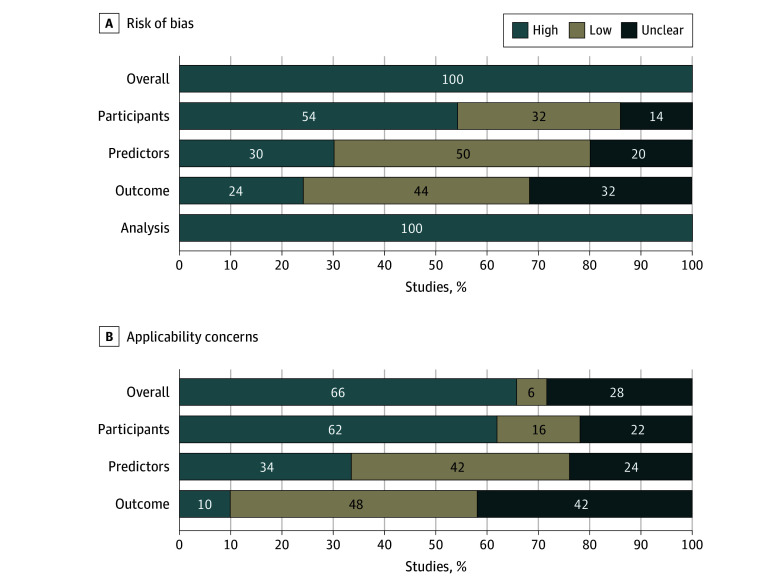
Risk of Bias and Applicability Concerns Risk of bias and applicability concerns were rated using the Prediction Model Risk of Bias Assessment Tool assessment tool as low, high, and unclear considering 4 domains for bias (A) and 3 for applicability (B). For bias and applicability, an overall rating is presented above the domain-specific ratings. See eTable 5 in Supplement 1 for details.

Risk of bias and applicability assessment are summarized in Figure 2 and detailed in eTable 6 in Supplement 1. All included studies were at a high risk of bias due to inadequate selection of study population (27 studies [54%]^26,27,28,33,37,41,43,44,45,47,48,51,52,53,55,56,59,60,62,64,65,66,67,68,69,71,75^), shortcomings in methods of measurement of predictors (15 studies [30%]^26,33,37,48,51,52,57,58,59,61,62,63,64,67,72^) and outcome (12 studies [24%]^28,33,38,45,48,53,54,55,58,59,65,67^), and flaws in the analysis strategy (all studies [100%]^26,27,28,29,30,31,32,33,34,35,36,37,38,39,40,41,42,43,44,45,46,47,48,49,50,51,52,53,54,55,56,57,58,59,60,61,62,63,64,65,66,67,68,69,70,71,72,73,74,75^). Concerns for applicability were also high, as study participants (31 studies [62%]^26,27,28,33,36,37,41,43,44,45,47,48,51,52,55,56,57,58,59,60,62,63,64,65,66,67,68,69,70,71,75^), predictors (17 studies [34%]^26,33,40,48,51,52,56,57,58,59,62,63,64,67,69,70,72^), and outcome (5 studies [10%]^28,48,58,65,67^) did not fit the intended target clinical setting.

## Discussion

This systematic review identified 50 mortality prediction models for people with kidney failure published in the last 2 decades. All models were created using data from people with kidney failure receiving hemodialysis or peritoneal dialysis. No study included people with kidney failure before they had made a treatment decision. While some models were created with a well-designed prediction framework, thus far none demonstrated promise in terms of clinical usability or incorporation into guidelines. None of the prediction models were ready to be updated^77^ or retrained.^24^ New research is needed to create clinical prediction models for people with kidney failure before and after a treatment decision has been made.

A recent meta-analysis^78^ completed a quantitative comparison of mortality prediction performance measures obtained from studies reporting mortality prediction models developed for patients starting dialysis. However, model performance measures do not have a direct clinical use, and the meta-analysis did not propose a new prediction model. Hence the value of meta-analytic summaries of prediction performance is limited.^18^ Our review focused on the quality and appropriateness of existing prediction models with emphasis on risk of bias and applicability concerns as defined by the CHARMS and PROBAST tools.^12,14,17^ According to these reporting guidelines, existing prediction models that this review identified should not be used in clinical practice without further evaluation.

Most studies included in this review focused on measures of discrimination for the evaluation of their prediction models. The most common measures of discrimination for survival analyses are the time-dependent AUC and the C index.^20^ Both measures assess the ability of the prediction model conditional on the outcome, and hence, they have limited clinical value because the outcome is unknown at the prediction time origin when treatment decisions are made. Calibration, on the other hand, has a direct clinical interpretation. For example, if a well-calibrated model predicts that the 5-year risk of death for a patient is 27%, then we can expect that 27 of 100 persons similar to that patient will die within 5 years.^18^ Note also that a model can systematically predict too high (or too low) risks, and the discrimination measures may not detect this bias (miscalibration).

Mortality prediction models for people with kidney failure can support patient-clinician discussions at a crucial point where decisions are made about treatment options, including long-term kidney replacement therapy, conservative kidney management, and/or end-of-life planning.^7^ A predicted risk can guide people with kidney failure when they engage in informed discussions on prognosis, decision-making, and life planning according to their personal views and values. Existing guidelines and the American Society of Nephrology Choosing Wisely campaign emphasize that individualized prognostic information should be included in the decision to initiate dialysis.^79^ However, current CKD guidelines do not recommend the use of any specific prediction model for mortality.^8^ Our review identified an abundance of mortality prediction models for people with kidney failure, suggesting that limitations in study design rather than number of studies is the primary problem. None of the existing studies involved patients, caregivers, and clinicians in prediction model design, which may help to promote acceptability and enhance usability and uptake in clinical practice. End-user engagement is key to ensure that the needs and personal preferences are addressed when a prediction model is created starting with the target population, including people who are making treatment decisions about dialysis, conservative kidney management, and/or dialysis withdrawal.

Our study has implications for future research. New mortality prediction models should be created for people with kidney failure. To inform the choice of conservative kidney management vs dialysis, the study cohort should include people with kidney failure before the treatment has been decided. The prediction time origin should be set at the time when treatment decisions are made. Predictors should be measured up to the prediction time origin and not after. If updated information on predictors or treatments is available,^80^ eg, after 6 months from the initial prediction origin, an updated predicted risk can be provided for people who have survived the first 6 months. Key elements of the analysis plan for the creation of a medical prediction model include the preparation of a learning dataset that reflects the prediction framework, the choice of an adequate scoring rule to compare rival modeling strategies, and some form of cross-validation to evaluate the prediction performance of the final model. External testing could be performed using temporally or geographically distinct data considering that heterogeneity in model performance across time and geography is expected due to differences in patient characteristics, clinical practice, health policy, and measurement procedures across regions and over time within the same region.^76^ Temporal testing works by splitting the study cohort according to a calendar date into training and testing data. This is advised to mimic the performance of the model in future patients and to identify any deterioration in model performance due to population or health care practice changes over time. The model can subsequently be updated using more recent data and its reproducibility verified as new temporally distinct data become available. Geographical testing for transportability is useful if the target populations and settings do not deviate too much from those for which the model was originally designed. Finally, the model should be evaluated in real-life conditions, eg, by a cluster randomized study that compares treatment uptake and outcomes between people who use standard care and people who use a care strategy informed by the model.^25^

### Strengths and Limitations

Our study is strengthened by a comprehensive and sensitive search strategy that covered several databases without language restrictions. We used the CHARMS and PROBAST tools, which were specifically created for the critical appraisal of prediction models designed to inform treatment decisions.

Our study also has limitations, largely related to the limitations of the included studies. First, included prediction models were likely designed to address a range of different research needs, as opposed to inform clinical practice. Second, none involved people with kidney failure who still had to make treatment decisions or who chose conservative care without dialysis. Additionally, many studies included in this review were published before recommended reporting standards and quality assessment tools for risk prediction modeling were published.

## Conclusions

According to this systematic review of 50 studies, published mortality prediction models for people with kidney failure were not found suitable to inform clinical decision-making. We advocate for the use of existing guidelines and checklists to design, conduct, and report prediction modeling studies and the involvement of stakeholders in the study design to enhance model usability and clinical uptake.

## Notes

Supplement 1. **eMethods.** Detailed Methods **eTable 1.** MEDLINE, Embase, and Cochrane Search Strategies **eTable 2.** CHARMS Checklist **eTable 3.** PROBAST Signaling Questions **eTable 4.** TRIPOD+AI Checklist **eTable 5.** Prediction Framework, Model Training and Testing, and Usefulness for the Included Studies **eTable 6.** Characteristics of the Studies Included in the Systematic Review and Critical Appraisal for Risk of Bias and Applicability According to PROBAST **eAppendix.** Study Protocol
eReferences.

Supplement 2.
Data Sharing Statement
