# Supplementary material for: Mortality Risk Prediction Models for People With Kidney Failure: A Systematic Review
Source: JAMA Netw Open. 2025 Jan 3;8(1):e2453190. doi: 10.1001/jamanetworkopen.2024.53190 (PMC11699530; doi:10.1001/jamanetworkopen.2024.53190)
Supplement: Supplement 1. — eMethods. Detailed Methods eTable 1. MEDLINE, Embase, and Cochrane Search Strategies eTable 2. CHARMS Checklist eTable 3. PROBAST Signaling Questions eTable 4. TRIPOD+AI Checklist eTable 5. Prediction Framework, Model Training and Testing, and Usefulness for the Included Studies eTable 6. Characteristics of the Studies Included in the Systematic Review and Critical Appraisal for Risk of Bias and Applicability According to PROBAST eAppendix. Study Protocol eReferences. [file jamanetwopen-e2453190-s001.pdf]

Supplemental Online Content

Jarrar F, Pasternak M, Harrison TG, et al. Mortality risk prediction models for people with kidney failure. *JAMA Netw Open.* 2025;8(1):e2453190. doi:10.1001/jamanetworkopen.2024.53190

eMethods. Detailed Methods

eTable 1. MEDLINE, Embase, and Cochrane Search Strategies

eTable 2. CHARMS Checklist

eTable 3. PROBAST Signaling Questions

eTable 4. TRIPOD+AI Checklist

eTable 5. Prediction Framework, Model Training and Testing, and Usefulness for the Included Studies

eTable 6. Characteristics of the Studies Included in the Systematic Review and Critical Appraisal for Risk of Bias and Applicability According to PROBAST

eAppendix. Study Protocol

eReferences.

This supplemental material has been provided by the authors to give readers additional information about their work.

eMethods. Detailed Methods

This systematic review was conducted according to the guidelines set out in the CHecklist for critical Appraisal and data extraction for systematic Reviews of prediction Modelling Studies (CHARMS)[1] and the Prediction model Risk Of Bias Assessment Tool (PROBAST).[2,3] The CHARMS checklist provides guidance for formulating the review question, and for extracting data from the primary studies reporting prediction models. The PROBAST tool includes four domains: participants, predictors, outcome, and analysis. For each domain the tool provides signaling questions for determining whether the risk of bias and the applicability should be graded as low, high or unclear. This systematic review was reported according to the Transparent Reporting of multivariable prediction models for Individual Prognosis Or Diagnosis reporting guideline for systematic reviews of multivariable prediction models (TRIPOD-SRMA).[4] TRIPOD-SRMA contains 26 items and has superseded previous reporting guidelines.

We planned to conduct a systematic review without meta-analysis.

Systematic review registration
https://www.crd.york.ac.uk/prospero/display_record.php?ID=CRD42023486220
Registration date: December 5, 2023, last update October 10, 2024

Review question
What is the quality and clinical applicability of existing mortality prediction models for people with kidney failure?

Searches and sources
An information specialist and medical librarian (D.L.) searched Ovid MEDLINE, Ovid EMBASE, and the Cochrane Library from 2004, when the Kidney Disease: Improving Global Outcomes (KDIGO) was originally established to develop and implement guidelines for the care of people with kidney disease, to September 30, 2024. Searches combined terms from three concepts: 1) chronic kidney failure (e.g., CKD, renal insufficiency); 2) mortality (e.g., mortality, death) and 3) prediction modelling (e.g., calibration, measures of discrimination) and recommended filters for prediction models.[3] Terms were searched as keywords and subject headings (e.g., MEDLINE MeSH). No language restrictions were applied to the search strategy. A complete description of the search strategy is provided in the supplement (**eTable 1**). The reference list of all included articles was also searched for any additional, relevant articles. Study citations and abstracts were uploaded to Covidence to aid in study screening.

Study eligibility criteria
We included prospective and retrospective cohort studies that created or evaluated (with internal or external testing) mortality prediction models for people with kidney failure not yet treated with dialysis, choosing conservative care for kidney failure or commencing any form of dialysis (i.e., incident hemodialysis or peritoneal dialysis), with the outcome of all-cause mortality and at least 3 months of prediction time horizon. Letters, editorials, narrative reviews, commentaries, and case reports were excluded. Also, prediction models for people who received a kidney transplant were excluded due to their improved prognosis after kidney transplantation.

Condition or domain being studied
Kidney failure, defined as eGFR of less than 15 ml/min/1.73 m^2 sustained for more than 90 days or requirement for kidney replacement with long-term dialysis (hemodialysis or peritoneal dialysis) or transplantation.

Participants/population
People with kidney failure who were or were not yet treated for end-stage kidney failure at the study entry date. Treatment included conservative care for kidney failure or dialysis. Transplant patients have better prognosis and thus studies including exclusively transplant patients were excluded. Patients were considered 'incident' dialysis patients if the study entry date (prediction time origin) was the dialysis initiation date. Patients were considered 'prevalent' dialysis patients if they had been already on dialysis when they entered the study.

Main outcome
Mortality, from specific causes and all-cause mortality.

Data extraction
Two reviewers (F.J. and M.P.) independently screened all abstracts of studies based on titles and abstracts, and then reviewed full texts in a second stage of the review to determine eligibility based on the study eligibility criteria described above.
Reviewers used the CHARMS (**eTable 2**) and the PROBAST (**eTable 3**) checklists to extract data on study design, including study characteristics, prediction framework, model design, selection, training, and testing strategies, prediction performance measures, and applicability. Reviewers also considered the extent to which each study adhered to the TRIPOD+AI reporting standard for all prediction models, irrespective of whether regression or machine learning methods were used.[5] Discrepancies between the two reviewers in study selection for inclusion and data extraction were resolved by discussing with two arbitrators (P.R. and P.L.).
The following study characteristics were pre-specified and extracted for each study (CHARMS items): author, publication year, country, setting, data source, study design, number of participants, study start year, duration of follow-up, inclusion criteria, exclusion criteria, dialysis modality (if any), incident vs. prevalent dialysis proportion, primary predictors and primary outcome. We also independently extracted all the elements of the study prediction framework; these included target population, time origin, prediction time horizon, target of the analysis (individual risk of death), predictive variables, competing risks, model users and model evaluation (internal and external). See **eTable 2** for CHARMS checklist items.

Risk of bias (quality) assessment
We followed the PROBAST recommendations to evaluate the risk of bias and the applicability of included studies. Bias is the presence of systematic error in a study that may lead to distorted or flawed results hampering the study's internal validity. Prediction models are at high risk of bias when there are shortcomings in study design, conduct, or analysis that could lead to systematic bias in the predicted risks or in the estimates of a model's predictive performance. Applicability concerns of a model arise when the study that developed the model defined or captured data on population, predictors, or outcomes in ways that do not reflect the clinical setting where the model is intended to be used (target population, type and timing of measurement of the predictors and outcome). See **eTable 3** for PROBAST signaling questions.

© 2025 Jarrar F et al. *JAMA Network Open.*

Risk prediction framework
A medical risk prediction model should be developed in a framework that defines the target population, the prediction time origin (when the model is applied to predict the risk of a new patient), the prediction time horizon, the predictors, the outcome, and competing events (if any). The framework implicitly defines who can use the model, how and when.[6,7]

Modelling algorithm and evaluation of prediction performance
There are many different ways to specify a regression model, and also many ways to tune a machine algorithm such as a random forest or a neural network. By modelling algorithm we mean all data dependent steps of modelling, including data dependent selection of predictor variables and data dependent tuning of hyperparameters, that are needed to produce the prediction model based on a dataset. For the resulting prediction model to be useful for new patients, in particular to avoid bias due to overfitting, it is crucial that the modelling algorithm is tested with some form of data-splitting. Cross-validation simulates the real-life situation where the model is trained using a dataset (training data) and then applied to inform new patients about the risk of the outcome. Most cross-validation approaches repeatedly split the data into non-overlapping training and test datasets. The procedure applies all steps of the modelling algorithm, including variable selection and tuning of hyperparameters, using the training dataset, and then evaluates the prediction performance in the test dataset.[7] For the review we assessed the modeling algorithms that the primary studies used to make the prediction model. We considered modeling algorithms inappropriate if they based the selection of the predictors on univariate analyses and then used expert guided model building and goodness of fit testing to obtain the prediction model.[2,3] We also considered backward variable selection inappropriate,[8] unless the modeling algorithm was evaluated using cross-validation where all steps of the modelling algorithm, including the backward selection, were repeated in training sets and evaluated in independent test sets.
Reviewers assessed whether the primary study used a single split of the data or cross-validation for model testing using the learning data (internal testing). A single random split is not recommended because the results will typically depend on the random seed (Monte-Carlo error), it is prone to manipulation and conceals part of the learning data.[7] Finally, we assessed whether the final model was evaluated using data the model had not seen during learning (external testing based on temporally or geographically distinct data). Of note, we intentionally avoided the use of the term 'validation' throughout this reporting,[9] as any statistical model falls short of the complexities of reality and thus cannot ultimately be considered valid. Instead, whether a prediction model is useful (or potentially harmful) should ultimately be tested in a randomized trial.[10]

Prediction models and key performance measures
Studies that follow people until death are characterized by right censored data. The reviewers evaluated if authors had used appropriate statistical methods for right censored data and provided details on how censoring was handled (see **eTable 3**, PROBAST, Analysis domain, signaling question 6).
Reviewers extracted the criteria for model evaluation and comparison of rival models, including calibration plots and the time-dependent area under the receiver operating characteristic curve, AUC, a measure of discrimination, and the time-dependent Brier score or prediction error, a measure of both calibration and discrimination.[7] Reviewers also reported if the primary study had used improper performance measures, including the c-index (Harrell concordance index)[11] and measures of reclassification.[12,13] These measures are not proper because they may erroneously show that a mis-specified model systematically outperforms the data-generating model.[7]

© 2025 Jarrar F et al. *JAMA Network Open.*

Other criteria for model evaluation
Reviewers assessed whether study authors applied decision curve analysis,[14] and whether they linked specific clinical decisions with categories defined by the predicted risks.[15] Finally, reviewers considered whether a model was tested in a clinical trial, as this is the ultimate test of model utility.[10] For usability, reviewers noted whether authors provided a calculator, a nomogram, or an alternative tool that would ease the access to patient predicted risks.

© 2025 Jarrar F et al. *JAMA Network Open.*

eTable 1. MEDLINE, Embase, and Cochrane Search Strategies

MEDLINE (2004 to September 30, 2024)	EMBASE (2004 to September 30, 2024)	Cochrane (2004 to September 30, 2024)
1 renal insufficiency, chronic/ or kidney failure, chronic/<br>2 ((chronic or end stage or stage 5) adj3 (renal or kidney*)).tw,kf.<br>3 (ckd or esrd or eskd).tw,kf.<br>4 1 or 2 or 3	1 chronic kidney failure/<br>2 ((chronic or end stage or stage 5) adj3 (renal or kidney*)).tw,kf.<br>3 (ckd or esrd or eskd).tw,kf.<br>4 1 or 2 or 3	1 MeSH descriptor: [Renal Insufficiency, Chronic] this term only<br>2 MeSH descriptor: [Kidney Failure, Chronic] this term only<br>3 (((chronic or end stage or stage 5) NEAR/3 (renal or kidney*))):ti,ab,kw (Word variations have been searched)<br>4 ((ckd or esrd or eskd)):ti,ab,kw (Word variations have been searched)
5 (death or mortality or survival).tw,kf.<br>6 area under curve/<br>7 (area under adj2 (curve or roc or receiver)).tw,kf.<br>8 (AUC or AUROC or accura* or brier score* or c-index or c-statistic or calibrat* or discriminat* or E-O ratio* or expected ratio* or Hosmer-Lemeshow or H-L test* or net reclassification or observed ratio*).tw,kf.<br>9 6 or 7 or 8	5 (death or mortality or survival).tw,kf.<br>6 exp area under the curve/<br>7 (area under adj2 (curve or roc or receiver)).tw,kf.<br>8 (AUC or AUROC or accura* or brier score* or c-index or c-statistic or calibrat* or discriminat* or E-O ratio* or expected ratio* or Hosmer-Lemeshow or H-L test* or net reclassification or observed ratio*).tw,kf.<br>9 6 or 7 or 8	5 #1 OR #2 OR #3 OR #4<br>6 ((death or mortality or survival)):ti,ab,kw (Word variations have been searched)<br>7 #5 AND #6<br>8 MeSH descriptor: [Area Under Curve] this term only<br>9 ((area under NEAR/2 (curve or roc or receiver))):ti,ab,kw (Word variations have been searched)
10 (equation* or model* or risk* or rule* or scor* or tool*).tw,kf.<br>11 9 and 10	10 (equation* or model* or risk* or rule* or scor* or tool*).tw,kf.<br>11 9 and 10	10 ((AUC or AUROC or accura* or brier score* or c-index or c-statistic or calibrat* or discriminat* or E-O ratio* or expected ratio* or Hosmer-Lemeshow or H-L test* or net reclassification or

© 2025 Jarrar F et al. *JAMA Network Open.*

		observed ratio*)):ti,ab,kw (Word variations have been searched)<br>11 8 OR 9 OR 10
12 ((predict* or prognostic* or risk) adj3 (algorithm* or equation* or index or model* or rule* or scor* or stratification or tool*)).tw,kf.<br>13 11 or 12<br>14 4 and 5 and 13	12 ((predict* or prognostic* or risk) adj3 (algorithm* or equation* or index or model* or rule* or scor* or stratification or tool*)).tw,kf.<br>13 11 or 12<br>14 4 and 5 and 13	12 ((equation* or model* or risk* or rule* or scor* or tool*)):ti,ab,kw (Word variations have been searched)<br>13 #11 AND #12<br>14 (((predict* or prognostic* or risk) NEAR/3 (algorithm* or equation* or index or model* or rule* or scor* or stratification or tool*))):ti,ab,kw (Word variations have been searched)
15 animals/ not humans/<br>16 14 not 15	15 animals/ not human/<br>16 14 not 15	15 #13 OR #14<br>16 #7 AND #15
17 limit 16 to yr="2005 -Current"<br>18 limit 17 to (case reports or comment or editorial or letter)<br>19 17 not 18	17 limit 16 to yr="2005 -Current"<br>18 limit 17 to (books or chapter or conference abstract or editorial or letter)<br>19 17 not 18	17 limit to 2005

© 2025 Jarrar F et al. *JAMA Network Open.*

eTable 2. CHARMS Checklist

Items guiding the review aim, search and study eligibility and prediction framework
**P**opulation: target population, people with kidney failure (CHARMS item 4)
**I**ndex: model development with/without external testing (CHARMS items 1 and 3)
**C**omparator: possible existing alternative models (CHARMS item 1)
**O**utcome: mortality, time from disease onset to death (CHARMS item 5)
**T**ime frame: from disease onset (origin) to any (pre-specified) prediction horizon, e.g., one year from initiation of dialysis (CHARMS items 6 and 7)
**S**etting: settings and intended use, e.g. people with kidney failure who need to make treatment (conservative care or dialysis) or end of life decisions (CHARMS item 2)
Items to extract from each study
1. Source of data: registry, administrative data, clinical trial or cohort study
2. Participants: people who develop kidney failure, defined as residual kidney function below a pre-specified threshold (eGFR <15 ml/min/1.73 m2) or initiation of kidney replacement, type of treatment (hemodialysis or peritoneal dialysis), recruitment method, number of centers, study dates
3. Outcome definition and determination: data sources, method of measurement, consistency and completeness, assessment without knowledge of the predictors
4. Predictors: number and type, timing of measurement (before or at time origin), assessment blinded for outcome, handling of linear predictors
5. Sample size (power): Number of participants and number of outcomes/events in relation to the number of candidate predictors
6. Missing data: Number of participants with any missing value for predictors or outcomes, handling of missing data (e.g., complete-case analysis, imputation,<br>or other methods)
7. Model development: MODELLING METHOD: regression or machine learning techniques, given the nature of the data (right censored observations) methods that do not use survival analysis need to be justified; MODEL SELECTION: criteria for final model design and choice, including shrinkage
8. Model performance: calibration plot, measures of discrimination with confidence intervals

© 2025 Jarrar F et al. *JAMA Network Open.*

9. Model evaluation: internal testing (resampling methods, bootstrapping and cross-validation, recommended; random split, not recommended) and external testing (temporally or geographically distinct data); if the model was adjusted or updated, methods used for evaluation (*note: an adjusted model is a new model*)
10. Results: final model summary (regression coefficients, hyperparameters of a machine learning model) and performance measures (with confidence intervals); any alternative representation of the model (nomogram, score chart, etc.), distribution of predictors and missing data in different datasets
11. Interpretation: conclusion of the authors, comparison with other studies

Legend: note that we followed the PICOTS definitions according to PROBAST, which include the original CHARMS items.

© 2025 Jarrar F et al. *JAMA Network Open.*

eTable 3. PROBAST Signaling Questions

Participants** – domain assessed for **risk of bias** and **applicability
1. Were appropriate data sources used, e.g., cohort, RCT, or nested case–control study data?
2. Were all inclusions and exclusions of participants appropriate?
Predictors** – domain assessed for **risk of bias** and **applicability
1. Were predictors defined and assessed in a similar way for all participants?
2. Were predictor assessments made without knowledge of outcome data?
3. Are all predictors available at the time the model is intended to be used?
Outcome** – domain assessed for **risk of bias** and **applicability
1. Was the outcome determined appropriately?
2. Was a prespecified or standard outcome definition used?
3. Were predictors excluded from the outcome definition?
4. Was the outcome defined and determined in a similar way for all participants?
5. Was the outcome determined without knowledge of predictor information?
6. Was the time interval between predictor assessment and outcome determination appropriate?
Analysis** – domain assessed for **risk of bias
1. Were there a reasonable number of participants with the outcome?
2. Were continuous and categorical predictors handled appropriately?
3. Were all enrolled participants included in the analysis?
4. Were participants with missing data handled appropriately?
5. Was selection of predictors based on univariable analysis avoided?†
6. Were complexities in the data (e.g., censoring, competing risks, sampling of control participants) accounted for appropriately?

© 2025 Jarrar F et al. *JAMA Network Open.*

7. Were relevant model performance measures evaluated appropriately?
8. Were model overfitting, underfitting, and optimism in model performance accounted for?†
9. Do predictors and their assigned weights in the final model correspond to the results from the reported multivariable analysis?†

Legend
RCT = randomized controlled trial; ROB = risk of bias.
* For further details, please see the explanation and elaboration document (27), available at Annals.org, and www.probast.org. Signaling questions
are answered as yes, probably yes, probably no, no, or no information. ROB and concerns for applicability are rated as low, high, or unclear.
† Development studies only.

Overall rating for each study considers 20 signaling questions, evaluator judgment and rationale for the judgment. Signaling questions were answered as "yes," "probably yes," "probably no," "no," or "no information", whereby "yes" indicates absence of bias and no applicability concern and "no" indicates presence of bias or applicability concern. After answering signaling questions, assessors then independently used their own judgments to determine whether the domain should be rated as high, low, or unclear risk of bias.

eTable 4. TRIPOD+AI Checklist

A. Title and Abstract (1-2), Introduction (3a-4), and Methods (5a-11) sections

Study	1	2	3a	3b	3c	4	5a	5b	6a	6b	6c	7	8a	8b	8c	9a	9b	9c	10	11
Chen, 2014[16]	Yes	Yes	Yes	No	No	Yes	Yes	Yes	No	Yes	NA	No	Yes	No	NA	Yes	Yes	No	No	No
Obi, 2018[17]	Yes	Yes	Yes	Yes	No	Yes	Yes	Yes	Yes	Yes	NA	No	Yes	No	NA	Yes	Yes	No	No	Yes
Inaguma, 2019[18]	Yes	Yes	Yes	Yes	No	Yes	Yes	Yes	Yes	Yes	NA	No	Yes	No	NA	Yes	Yes	No	No	No
Santos, 2020[19]	Yes	Yes	Yes	No	No	Yes	Yes	Yes	Yes	Yes	NA	No	Yes	No	NA	No	Yes	No	No	No
Pladys, 2020[20]	Yes	Yes	Yes	Yes	No	Yes	Yes	Yes	Yes	Yes	NA	No	Yes	No	NA	Yes	Yes	No	No	Yes
Hemke, 2013[21]	No	Yes	Yes	Yes	No	Yes	Yes	Yes	Yes	Yes	NA	No	Yes	No	NA	Yes	Yes	No	No	Yes
Chen, 2017[22]	No	Yes	Yes	Yes	No	Yes	Yes	Yes	Yes	Yes	NA	No	Yes	No	NA	No	No	No	No	Yes
Chua, 2014[23]	Yes	Yes	Yes	Yes	No	Yes	Yes	Yes	Yes	Yes	NA	No	Yes	No	NA	No	Yes	No	No	No
van Dieppen, 2014[24]	Yes	Yes	Yes	Yes	No	Yes	Yes	Yes	Yes	Yes	NA	No	Yes	No	NA	Yes	Yes	No	No	Yes
Floege, 2015[25]	Yes	Yes	Yes	Yes	No	Yes	Yes	Yes	Yes	Yes	NA	No	Yes	NA	NA	No	Yes	No	No	Yes
Dusseaux, 2015[26]	No	Yes	Yes	Yes	No	Yes	Yes	Yes	Yes	Yes	NA	No	Yes	No	NA	No	Yes	No	No	Yes
Doi, 2015[27]	Yes	Yes	Yes	Yes	No	Yes	Yes	Yes	Yes	Yes	NA	No	Yes	No	NA	Yes	Yes	No	No	Yes
Thamer, 2015[28]	Yes	Yes	Yes	Yes	No	Yes	Yes	Yes	Yes	Yes	NA	No	Yes	No	NA	No	Yes	No	No	Yes
Couchoud, 2015[29]	No	Yes	Yes	Yes	No	Yes	Yes	Yes	Yes	Yes	NA	No	Yes	No	NA	No	Yes	No	No	Yes
Hemke, 2015[30]	Yes	Yes	Yes	Yes	No	Yes	Yes	Yes	Yes	Yes	NA	No	Yes	No	NA	Yes	Yes	No	No	Yes
Patzer, 2016[31]	No	Yes	Yes	Yes	No	Yes	Yes	Yes	Yes	Yes	NA	No	Yes	No	NA	Yes	Yes	No	No	Yes

© 2025 Jarrar F et al. *JAMA Network Open.*

Haapio, 2017[32]	Yes	Yes	Yes	Yes	No	Yes	Yes	Yes	Yes	Yes	NA	No	Yes	No	NA	Yes	Yes	No	No	Yes
Lin, 2019[33]	Yes	No	Yes	Yes	No	Yes	No	Yes	Yes	No	NA	No	Yes	No	NA	No	No	No	No	No
Akbilgic, 2019[34]	No	Yes	Yes	Yes	No	Yes	Yes	Yes	Yes	Yes	NA	No	Yes	No	NA	No	Yes	No	No	Yes
Cho, 2017[35]	No	Yes	Yes	Yes	No	Yes	No	Yes	No	Yes	NA	No	Yes	No	NA	Yes	Yes	No	No	No
Wick, 2017[36]	Yes	Yes	Yes	Yes	No	Yes	Yes	Yes	Yes	Yes	NA	No	Yes	No	NA	No	Yes	No	No	No
Ivory, 2017[37]	Yes	Yes	Yes	Yes	No	Yes	Yes	Yes	Yes	Yes	NA	No	Yes	No	NA	No	Yes	No	No	Yes
Geddes, 2006[38]	No	Yes	Yes	Yes	No	Yes	Yes	Yes	Yes	Yes	NA	No	Yes	No	NA	No	Yes	No	No	Yes
Mauri, 2008[39]	Yes	Yes	Yes	Yes	No	Yes	Yes	Yes	Yes	Yes	NA	No	Yes	No	NA	No	Yes	No	No	No
Couchoud, 2009[40]	Yes	Yes	Yes	Yes	No	Yes	Yes	Yes	Yes	Yes	NA	No	Yes	No	NA	Yes	Yes	No	No	Yes
Liu, 2010[41]	Yes	Yes	Yes	No	No	Yes	Yes	Yes	Yes	Yes	NA	No	Yes	No	NA	No	Yes	No	No	No
Jacob, 2010[42]	Yes	Yes	No	No	No	No	Yes	Yes	Yes	Yes	NA	No	No	No	NA	No	Yes	No	No	Yes
Marinovich, 2010[43]	Yes	Yes	Yes	Yes	No	Yes	Yes	Yes	Yes	Yes	NA	No	Yes	No	NA	No	Yes	No	No	No
Quinn, 2011[44]	Yes	Yes	Yes	Yes	No	Yes	Yes	Yes	Yes	Yes	NA	No	Yes	No	NA	Yes	Yes	No	No	No
Wu, 2022[45]	Yes	No	Yes	Yes	No	Yes	Yes	Yes	Yes	Yes	NA	No	Yes	No	NA	No	No	No	No	Yes
Noh, 2020[46]	Yes	Yes	Yes	No	No	Yes	Yes	Yes	Yes	Yes	NA	No	Yes	No	NA	No	No	No	No	Yes
Siddiqa, 2021[47]	Yes	Yes	Yes	Yes	No	Yes	Yes	Yes	Yes	Yes	NA	No	Yes	No	NA	No	No	No	No	No
McAdams-DeMarco, 2018[48]	No	Yes	Yes	No	No	Yes	Yes	Yes	Yes	Yes	NA	No	Yes	No	NA	No	No	No	No	No
Gao, 2022[49]	Yes	Yes	Yes	No	No	Yes	Yes	Yes	Yes	Yes	NA	No	Yes	No	NA	No	No	No	No	No
Chaudhuri, 2023[50]	No	Yes	Yes	No	No	Yes	Yes	Yes	Yes	Yes	NA	No	Yes	No	NA	No	No	No	No	No
Thijssen, 2012[51]	Yes	Yes	Yes	No	No	Yes	Yes	Yes	Yes	Yes	NA	No	Yes	No	NA	No	Yes	No	No	No

© 2025 Jarrar F et al. *JAMA Network Open.*

Wagner, 2011[52]	Yes	Yes	Yes	Yes	No	Yes	Yes	Yes	Yes	Yes	NA	No	Yes	No	NA	Yes	Yes	No	No	No
Zhu, 2021[53]	Yes	Yes	Yes	No	No	Yes	Yes	Yes	Yes	No	NA	No	Yes	No	NA	No	No	No	No	No
Cohen, 2010[54]	Yes	Yes	Yes	Yes	No	Yes	Yes	Yes	Yes	No	NA	No	Yes	No	NA	Yes	No	No	No	Yes
Siga, 2020[55]	Yes	Yes	Yes	No	No	Yes	Yes	Yes	Yes	No	NA	No	Yes	No	NA	No	No	No	No	Yes
Jung, 2018[56]	Yes	Yes	Yes	No	No	Yes	Yes	Yes	Yes	Yes	NA	No	Yes	No	NA	No	No	No	No	No
Wang, 2021[57]	Yes	Yes	Yes	No	No	Yes	Yes	Yes	Yes	Yes	NA	No	Yes	No	NA	No	No	No	No	No
Tapak, 2020[58]	No	Yes	Yes	No	No	Yes	Yes	Yes	Yes	Yes	NA	No	Yes	No	NA	No	No	No	No	No
Holme, 2012[59]	Yes	Yes	Yes	No	No	Yes	Yes	Yes	Yes	Yes	NA	No	Yes	No	NA	Yes	No	No	No	No
Rankin, 2022[60]	Yes	Yes	Yes	Yes	No	Yes	Yes	Yes	Yes	Yes	NA	No	Yes	No	NA	Yes	Yes	No	No	Yes
Fernandez Lucas, 2007[61]	Yes	Yes	Yes	Yes	No	Yes	No	Yes	No	No	NA	No	Yes	No	NA	No	Yes	No	No	No
Goldstein, 2024[62]	Yes	Yes	Yes	No	No	Yes	Yes	Yes	Yes	Yes	NA	No	Yes	No	NA	Yes	Yes	No	No	No
Noppakun, 2023[63]	Yes	Yes	Yes	Yes	No	Yes	Yes	Yes	Yes	Yes	NA	No	Yes	No	NA	Yes	Yes	No	No	No
Yang, 2023[64]	Yes	Yes	Yes	Yes	No	Yes	Yes	Yes	Yes	Yes	NA	No	Yes	No	NA	No	Yes	No	No	No
Okada, 2024[65]	Yes	Yes	Yes	Yes	No	Yes	Yes	Yes	Yes	Yes	NA	No	Yes	No	NA	No	Yes	No	No	Yes

Abbreviations: NA, not applicable. The section/topics for the numbered TRIPOD+AI checklist items can be found at: Collins GS et al. TRIPOD+AI statement: updated guidance for reporting clinical prediction models that use regression or machine learning methods.

© 2025 Jarrar F et al. *JAMA Network Open.*

B. Methods (12a-17) and Open Science (18a-f) sections

Study	12a	12b	12c	12d	12e	12f	12g	13	14	15	16	17	18a	18b	18c	18d	18e	18f
Chen, 2014[16]	No	Yes	Yes	No	Yes	NA	Yes	NA	No	Yes	Yes	Yes	Yes	No	No	No	No	NA
Obi, 2018[17]	Yes	Yes	Yes	No	Yes	NA	Yes	NA	No	Yes	Yes	Yes	Yes	Yes	No	No	Yes	NA
Inaguma, 2019[18]	No	No	No	No	Yes	NA	Yes	NA	No	Yes	No	Yes	Yes	Yes	No	Yes	Yes	NA
Santos, 2020[19]	No	Yes	Yes	No	Yes	NA	No	NA	No	Yes	Yes	Yes	No	Yes	No	No	No	NA
Pladys, 2020[20]	Yes	Yes	Yes	No	Yes	NA	Yes	NA	No	Yes	Yes	Yes	No	No	No	No	Yes	NA
Hemke, 2013[21]	Yes	Yes	Yes	No	Yes	NA	Yes	NA	No	Yes	Yes	Yes	No	No	No	No	No	NA
Chen, 2017[22]	Yes	Yes	Yes	No	Yes	NA	Yes	NA	No	Yes	Yes	No	Yes	Yes	No	No	Yes	NA
Chua, 2014[23]	No	Yes	Yes	No	Yes	NA	Yes	NA	No	Yes	NA	Yes	No	Yes	No	No	No	NA
van Dieppen, 2014[24]	No	Yes	Yes	No	Yes	NA	Yes	NA	No	No	NA	Yes	Yes	No	No	No	Yes	NA
Floege, 2015[25]	Yes	Yes	Yes	No	Yes	NA	Yes	NA	No	Yes	Yes	Yes	Yes	Yes	No	No	Yes	NA
Dusseaux, 2015[26]	Yes	Yes	Yes	No	Yes	NA	No	NA	No	Yes	Yes	No	Yes	Yes	No	No	Yes	NA
Doi, 2015[27]	No	Yes	Yes	No	Yes	NA	No	NA	No	No	Yes	Yes	Yes	Yes	No	Yes	No	NA
Thamer, 2015[28]	Yes	Yes	Yes	No	Yes	NA	No	NA	No	No	Yes	Yes	Yes	Yes	No	No	Yes	NA
Couchoud, 2015[29]	Yes	Yes	Yes	No	Yes	NA	No	NA	No	Yes	No	No	No	No	No	No	No	NA
Hemke, 2015[30]	Yes	Yes	Yes	No	Yes	Yes	Yes	NA	No	Yes	Yes	Yes	No	Yes	No	No	Yes	NA
Patzer, 2016[31]	Yes	Yes	Yes	No	Yes	NA	Yes	NA	No	Yes	Yes	Yes	Yes	Yes	Yes	No	Yes	NA
Haapio, 2017[32]	Yes	Yes	Yes	No	Yes	NA	Yes	NA	No	Yes	Yes	No	Yes	Yes	No	No	No	NA
Lin, 2019[33]	No	No	No	No	Yes	NA	No	No	No	No	No	Yes	Yes	Yes	No	No	Yes	No

© 2025 Jarrar F et al. *JAMA Network Open.*

Akbilgic, 2019[34]	Yes	Yes	Yes	No	Yes	Yes	Yes	No	No	Yes	NA	Yes	Yes	Yes	No	No	Yes	Yes
Cho, 2017[35]	Yes	Yes	Yes	No	Yes	Yes	Yes	NA	No	Yes	Yes	Yes	Yes	Yes	No	No	Yes	NA
Wick, 2017[36]	Yes	Yes	Yes	No	Yes	NA	No	NA	No	Yes	NA	Yes	Yes	Yes	No	No	Yes	NA
Ivory, 2017[37]	Yes	Yes	Yes	No	Yes	Yes	Yes	NA	No	Yes	No	Yes	Yes	Yes	No	No	No	NA
Geddes, 2006[38]	Yes	Yes	Yes	No	Yes	NA	Yes	NA	No	Yes	Yes	No	No	Yes	No	No	No	NA
Mauri, 2008[39]	Yes	Yes	Yes	No	Yes	NA	No	NA	No	Yes	No	No	No	Yes	No	No	No	NA
Couchoud, 2009[40]	Yes	Yes	Yes	No	Yes	NA	Yes	NA	No	Yes	Yes	No	No	Yes	No	No	No	NA
Liu, 2010[41]	Yes	Yes	Yes	No	Yes	NA	Yes	NA	No	Yes	Yes	No	No	Yes	No	No	No	NA
Jacob, 2010[42]	No	Yes	Yes	No	Yes	Yes	No	No	No	No	NA	No	Yes	No	No	No	Yes	No
Marinovich, 2010[43]	No	Yes	Yes	No	Yes	NA	No	NA	No	Yes	NA	No	No	Yes	No	No	No	NA
Quinn, 2011[44]	Yes	Yes	Yes	No	Yes	NA	Yes	NA	No	Yes	NA	Yes	Yes	No	No	No	Yes	NA
Wu, 2022[45]	Yes	No	No	No	Yes	NA	Yes	NA	No	No	Yes	No	Yes	Yes	No	No	Yes	NA
Noh, 2020[46]	No	Yes	Yes	No	Yes	No	Yes	No	No	Yes	No	Yes	Yes	Yes	No	No	Yes	No
Siddiqa, 2021[47]	No	Yes	Yes	No	Yes	No	Yes	NA	No	No	No	Yes	Yes	Yes	No	No	No	NA
McAdams-DeMarco, 2018[48]	No	Yes	Yes	No	Yes	NA	No	NA	No	No	NA	Yes	No	Yes	No	No	No	NA
Gao, 2022[49]	No	Yes	Yes	No	Yes	NA	No	NA	No	No	NA	Yes	Yes	Yes	No	No	Yes	NA
Chaudhuri, 2023[50]	Yes	Yes	Yes	No	Yes	NA	Yes	No	No	Yes	No	Yes	Yes	Yes	No	No	No	No
Thijssen, 2012[51]	Yes	Yes	Yes	No	Yes	No	Yes	NA	No	Yes	Yes	No	No	No	No	No	No	NA
Wagner, 2011[52]	Yes	Yes	Yes	No	Yes	No	Yes	NA	No	Yes	Yes	Yes	Yes	Yes	No	No	No	NA
Zhu, 2021[53]	No	Yes	Yes	No	Yes	NA	Yes	NA	No	Yes	NA	Yes	Yes	Yes	No	No	No	NA

© 2025 Jarrar F et al. *JAMA Network Open.*

Cohen, 2010[54]	Yes	Yes	Yes	No	Yes	No	Yes	NA	No	Yes	Yes	Yes	Yes	Yes	No	No	No	NA
Siga, 2020[55]	No	Yes	Yes	No	Yes	No	Yes	No	No	No	No	No	Yes	Yes	No	No	Yes	No
Jung, 2018[56]	No	Yes	Yes	No	Yes	NA	Yes	NA	No	No	NA	Yes	Yes	Yes	No	No	Yes	NA
Wang, 2021[57]	No	Yes	Yes	No	Yes	No	No	Yes	No	Yes	Yes	No	Yes	Yes	No	No	Yes	No
Tapak, 2020[58]	No	Yes	Yes	No	Yes	No	Yes	No	No	No	Yes	Yes	Yes	Yes	No	No	No	No
Holme, 2012[59]	No	Yes	Yes	No	Yes	No	Yes	NA	No	Yes	No	No	No	Yes	No	No	Yes	NA
Rankin, 2022[60]	Yes	Yes	Yes	No	Yes	NA	Yes	Yes	No	Yes	Yes	Yes	Yes	Yes	No	No	Yes	No
Fernandez Lucas, 2007[61]	No	Yes	Yes	No	Yes	NA	Yes	NA	No	Yes	NA	No	No	Yes	No	No	No	NA
Goldstein, 2024[62]	Yes	No	Yes	No	Yes	NA	Yes	No	No	Yes	No	Yes	Yes	Yes	No	No	No	No
Noppakun, 2023[63]	Yes	Yes	No	No	Yes	NA	No	NA	No	No	No	Yes	Yes	Yes	No	No	Yes	NA
Yang, 2023[64]	Yes	Yes	Yes	No	Yes	NA	No	NA	No	Yes	Yes	Yes	Yes	Yes	No	Yes	Yes	NA
Okada, 2024[65]	Yes	No	No	No	Yes	NA	No	NA	No	No	Yes	Yes	Yes	Yes	No	No	Yes	NA

Abbreviations: NA, not applicable. The section/topics for the numbered TRIPOD+AI checklist items can be found at: Collins GS et al. TRIPOD+AI statement: updated guidance for reporting clinical prediction models that use regression or machine learning methods.

© 2025 Jarrar F et al. *JAMA Network Open.*

C. Patient and Public Involvement (19), Results (20a-24), Discussion (25-27c) sections

Study	19	20a	20b	20c	21	22	23a	23b	24	25	26	27a	27b	27c
Chen, 2014[16]	No	Yes	Yes	NA	Yes	No	No	NA	NA	Yes	Yes	No	No	Yes
Obi, 2018[17]	No	Yes	Yes	Yes	Yes	Yes	Yes	NA	NA	Yes	Yes	No	No	Yes
Inaguma, 2019[18]	No	Yes	Yes	NA	Yes	No	Yes	NA	NA	Yes	Yes	No	No	Yes
Santos, 2020[19]	No	Yes	Yes	Yes	Yes	Yes	Yes	NA	NA	Yes	Yes	No	No	Yes
Pladys, 2020[20]	No	Yes	Yes	Yes	Yes	No	Yes	NA	NA	Yes	Yes	No	No	Yes
Hemke, 2013[21]	No	Yes	Yes	Yes	Yes	Yes	Yes	NA	NA	Yes	Yes	No	No	Yes
Chen, 2017[22]	No	No	Yes	Yes	Yes	No	No	NA	NA	Yes	Yes	No	No	Yes
Chua, 2014[23]	No	Yes	No	NA	Yes	Yes	Yes	NA	NA	Yes	Yes	No	No	Yes
van Dieppen, 2014[24]	No	No	Yes	NA	Yes	Yes	Yes	NA	NA	Yes	Yes	No	No	Yes
Floege, 2015[25]	No	Yes	Yes	Yes	Yes	Yes	Yes	NA	NA	Yes	Yes	No	No	Yes
Dusseaux, 2015[26]	No	Yes	Yes	Yes	Yes	No	Yes	NA	NA	Yes	Yes	No	No	Yes
Doi, 2015[27]	No	No	Yes	Yes	Yes	Yes	Yes	NA	NA	Yes	Yes	No	No	Yes
Thamer, 2015[28]	No	Yes	Yes	Yes	Yes	Yes	Yes	NA	NA	Yes	Yes	No	No	Yes
Couchoud, 2015[29]	No	No	Yes	No	Yes	Yes	Yes	NA	NA	Yes	Yes	No	No	Yes
Hemke, 2015[30]	No	Yes	Yes	Yes	Yes	Yes	No	NA	Yes	Yes	Yes	No	No	Yes
Patzer, 2016[31]	Yes	Yes	Yes	Yes	Yes	Yes	Yes	NA	NA	Yes	Yes	No	No	Yes
Haapio, 2017[32]	No	Yes	Yes	Yes	Yes	Yes	Yes	NA	NA	Yes	Yes	No	No	Yes
Lin, 2019[33]	No	No	No	No	No	No	Yes	NA	NA	Yes	Yes	No	No	Yes
Akbilgic, 2019[34]	No	Yes	Yes	NA	Yes	No	Yes	NA	NA	Yes	Yes	No	No	Yes

Cho, 2017[35]	No	Yes	No	Yes	Yes	No	Yes	NA	NA	Yes	Yes	No	No	Yes
Wick, 2017[36]	No	No	No	NA	Yes	No	No	NA	NA	Yes	Yes	No	No	Yes
Ivory, 2017[37]	No	Yes	Yes	No	Yes	Yes	Yes	NA	NA	Yes	Yes	No	No	Yes
Geddes, 2006[38]	No	Yes	Yes	Yes	Yes	Yes	Yes	NA	NA	Yes	Yes	No	No	Yes
Mauri, 2008[39]	No	No	No	No	Yes	Yes	Yes	NA	NA	Yes	Yes	No	No	Yes
Couchoud, 2009[40]	No	Yes	Yes	Yes	Yes	No	Yes	NA	NA	Yes	Yes	No	No	Yes
Liu, 2010[41]	No	No	No	Yes	Yes	No	Yes	NA	NA	Yes	Yes	No	No	Yes
Jacob, 2010[42]	No	No	No	No	Yes	No	Yes	NA	NA	Yes	No	No	No	Yes
Marinovich, 2010[43]	No	No	Yes	NA	Yes	No	Yes	NA	NA	Yes	Yes	No	No	No
Quinn, 2011[44]	No	No	No	No	Yes	No	Yes	NA	NA	Yes	Yes	No	No	Yes
Wu, 2022[45]	No	Yes	Yes	Yes	Yes	No	No	NA	NA	Yes	Yes	No	No	No
Noh, 2020[46]	No	Yes	Yes	No	Yes	No	Yes	NA	NA	Yes	No	No	No	Yes
Siddiqa, 2021[47]	No	No	No	No	No	No	No	NA	No	Yes	Yes	No	No	No
McAdams-DeMarco, 2018[48]	No	No	No	NA	Yes	Yes	Yes	NA	No	Yes	Yes	No	No	Yes
Gao, 2022[49]	No	Yes	Yes	NA	Yes	Yes	Yes	NA	No	Yes	Yes	No	No	Yes
Chaudhuri, 2023[50]	No	No	No	No	Yes	No	Yes	NA	NA	Yes	Yes	No	No	Yes
Thijssen, 2012[51]	No	Yes	No	Yes	Yes	Yes	Yes	NA	NA	Yes	Yes	No	No	Yes
Wagner, 2011[52]	No	Yes	Yes	Yes	Yes	Yes	Yes	NA	NA	Yes	Yes	No	No	Yes
Zhu, 2021[53]	No	No	No	NA	Yes	No	Yes	NA	NA	Yes	Yes	No	No	Yes
Cohen, 2010[54]	No	No	Yes	Yes	Yes	No	Yes	NA	NA	Yes	Yes	No	No	Yes
Siga, 2020[55]	No	Yes	Yes	No	Yes	No	Yes	NA	NA	Yes	Yes	No	No	Yes
Jung, 2018[56]	No	No	No	NA	Yes	No	Yes	NA	NA	Yes	Yes	No	No	Yes

© 2025 Jarrar F et al. *JAMA Network Open.*

Wang, 2021[57]	No	No	No	Yes	No	No	Yes	NA	NA	Yes	Yes	No	No	Yes
Tapak, 2020[58]	No	Yes	No	NA	Yes	No	Yes	NA	NA	Yes	Yes	No	No	Yes
Holme, 2012[59]	No	Yes	No	No	Yes	No	Yes	NA	NA	Yes	Yes	No	No	Yes
Rankin, 2022[60]	No	Yes	Yes	Yes	Yes	Yes	Yes	NA	Yes	Yes	Yes	No	No	Yes
Fernandez Lucas, 2007[61]	No	No	No	NA	Yes	No	Yes	NA	NA	Yes	Yes	No	No	No
Goldstein, 2024[62]	No	No	No	No	Yes	Yes	Yes	NA	NA	Yes	Yes	No	No	Yes
Noppakun, 2023[63]	No	Yes	No	No	Yes	No	No	NA	NA	Yes	Yes	No	No	Yes
Yang, 2023[64]	No	Yes	No	Yes	Yes	Yes	Yes	NA	NA	Yes	Yes	No	No	No
Okada, 2024[65]	No	Yes	Yes	Yes	Yes	No	Yes	NA	NA	Yes	Yes	No	No	Yes

Abbreviations: NA, not applicable. The section/topics for the numbered TRIPOD+AI checklist items can be found at: Collins GS et al. TRIPOD+AI statement: updated guidance for reporting clinical prediction models that use regression or machine learning methods.

© 2025 Jarrar F et al. *JAMA Network Open.*

eTable 5. Prediction Framework, Model Training and Testing, and Usefulness for the Included Studies

Author, Year	Target population	Time origin	Prediction time horizon	Model use	Model users	Internal testing	External testing	Discrimination	Calibration	Utility	Usability
Chen, 2014[16]	Yes	First dialysis	2.5 years	No	No	Yes; CV	No	Yes	Yes; plot	No	No
Obi, 2018[17]	Yes	First dialysis	3 months<br>6 months<br>9 months<br>12 months	Yes	Yes	Yes; SS	Yes; 2 datasets from same country (overlap?)	Yes	Yes; plot	No	Yes; web calculator
Inaguma, 2019[18]	Yes	First dialysis	12 months	Yes	Yes	Yes; BS	No	Yes	No	No	No
Santos, 2020[19]	Yes	First dialysis	6 months	Yes	No	Yes; BS	No	Yes	Yes; HL	No	Yes; score chart
Pladys, 2020[20]	Yes	First dialysis	12 months	Yes	Yes	Yes; SS	No	Yes	Yes; plot	No	Yes; web calculator
Hemke, 2013[21]	Yes	90-days	3 years<br>5 years<br>10 years	Yes	Yes	Yes; SS	No	Yes	Yes; plot	No	No
Chen, 2017[22]	Yes	First dialysis	5 years	No	Yes	Yes; SS	No	Yes	No	No	No
Chua, 2014[23]	Yes	First dialysis	3 months<br>12 months	Yes	Yes	No	No	Yes	Yes; HL	No	No

© 2025 Jarrar F et al. *JAMA Network Open.*

van Dieppen, 2014[24]	Yes	Unclear	12 months	Yes	Yes	Yes; BS	No	Yes	Yes; plot	No	Yes; risk calculator
Floege, 2015[25]	Yes	90-days	12 months<br>2 years	Yes	Yes	No	Yes; 2 datasets from different countries (overlap?)	Yes	Yes; plot	No	Yes; nomogra m
Dusseaux, 2015[26]	Yes	First dialysis	3 years	Yes	Yes	No	Yes; temporal	Yes	Yes; plot	No	No
Doi, 2015[27]	Yes	First dialysis	12 months	Yes	Yes	Yes; BS	No	Yes	Yes; plot	No	Yes; risk chart
Thamer, 2015[28]	Yes	First dialysis	3 months<br>6 months	Yes	Yes	No	Yes; temporal	Yes	Yes; plot	No	No
Couchoud, 2015[29]	Yes	First dialysis	3 months	Yes	Yes	Yes; SS	No	Yes	No	No	Yes; risk chart
Hemke, 2015[30]	Yes	90-days	3 years<br>5 years<br>10 years	Yes	Yes	Yes; SS	No	Yes	Yes; plot	No	No
Patzer, 2016[31]	Yes	First dialysis	12 months<br>3 years	Yes	Yes	Yes; SS	No	Yes	Yes; plot	No	Yes; risk calculator
Haapio, 2017[32]	Yes	First dialysis	12 months<br>2 years	Yes	Yes	No	Yes; temporal	Yes	Yes; plot	No	No
Lin, 2019[33]	Yes	Unclear	12 months	Yes	Yes	Yes; CV	No	Yes	Unclear	No	No
Akbilgic, 2019[34]	Yes	First dialysis	1 month<br>3 months	Yes	Yes	Yes; CV	No	Yes	No	No	No

© 2025 Jarrar F et al. *JAMA Network Open.*

			6 months<br>12 months								
Cho, 2017[35]	Yes	90-days	> 5 years	Yes	Yes	No	Yes; 2 datasets from same country (overlap?)	Yes	No	No	No
Wick, 2017[36]	Yes	First dialysis	6 months	Yes	Yes	Yes; CV	No	Yes	Yes; plot	No	Yes; risk calculator
Ivory, 2017[37]	Yes	First dialysis	6 months	Yes	Yes	No	Yes; temporal and geographic al	Yes	Yes; plot	No	Yes; risk chart
Geddes, 2006[38]	Yes	First dialysis	12 months<br>5 years	Yes	Yes	No	Yes; temporal	Yes	Yes; no plot	No	No
Mauri, 2008[39]	Yes	First dialysis	12 months	Yes	Yes	Yes; SS	No	Yes	Yes; no plot	No	No
Couchoud, 2009[40]	Yes	First dialysis	6 months	Yes	Yes	Yes; SS	No	Yes	Yes; plot	No	No
Liu, 2010[41]	Yes	Unclear	Unclear	No	No	No	Yes; temporal	Yes	No	No	No
Jacob, 2010[42]	Yes	First dialysis	6 months<br>9 months<br>12 months<br>2 years<br>3 years	Yes	No	Yes; SS	No	Yes	No	No	No

© 2025 Jarrar F et al. *JAMA Network Open.*

			4 years<br>5 years<br>>5 years								
Marinovich, 2010[43]	Yes	First dialysis	12 months	Yes	Yes	No	No	Yes	No	No	No
Quinn, 2011[44]	Yes	First dialysis	12 months	Yes	Yes	Yes; BS	No	Yes	Yes; plot	No	No
Wu, 2022[45]	Yes	First dialysis	3 months<br>12 months	Yes	Yes	Yes; CV	Yes; temporal	Yes	Yes; plot	No	Yes; nomogra m
Noh, 2020[46]	No	Unclear	5 years	No	No	Yes; SS	No	Yes	No	No	No
Siddiqa, 2021[47]	No	Unclear	5 years	No	Yes	Yes; SS	Yes; 2 datasets from same country (overlap?)	Yes	Yes; no plot	No	No
McAdams-DeMarco, 2018[48]	No	Unclear	Unclear	No	No	No	No	Yes	No	No	No
Gao, 2022[49]	No	Unclear	3 years<br>5 years	No	No	No	No	Yes	No	No	Yes; nomogra m
Chaudhuri, 2023[50]	No	Unclear	3 years	No	No	Yes; SS	No	Yes	No	No	No
Thijssen, 2012[51]	Yes	First dialysis	6 months	Yes	No	Yes; SS	No	Yes	No	No	No

© 2025 Jarrar F et al. *JAMA Network Open.*

Wagner, 2011[52]	Yes	Unclear	3 years	Yes	Yes	Yes; SS	No	Yes	Yes; plot	No	No
Zhu, 2021[53]	No	Unclear	3 years	No	No	No	No	Yes	Yes; plot	No	Yes; nomogra m
Cohen, 2010[54]	No	Unclear	1 year	No	Yes	No	Yes; temporal	Yes	No	No	No
Siga, 2020[55]	No	Unclear	2 years	No	No	Yes; CV	No	Yes	Yes; plot	No	No
Jung, 2018[56]	No	Unclear	1 year<br>3 years<br>5 years	No	No	No	No	Yes	Yes; plot	No	Yes; risk calculator
Wang, 2021[57]	No	Unclear	1 year	No	No	Yes; CV	No	Yes	No	No	No
Tapak, 2020[58]	No	Unclear	5 years	No	No	Yes; BS	No	Yes	No	No	No
Holme, 2012[59]	No	Unclear	3 years	No	No	Yes; SS	No	Yes	Yes; HL	No	No
Rankin, 2022[60]	Yes	First dialysis	3 months	Yes	Yes	Yes; SS	No	Yes	Yes; plot	No	No
Fernandez Lucas, 2007[61]	Yes	First dialysis	3 years	Yes	No	No	No	Yes	No	No	No
Goldstein, 2024[62]	Yes	First dialysis	Unclear	Yes	Yes	Yes; SS	No	Yes	Yes; plot	No	No
Noppakun, 2023[63]	Yes	First dialysis	3 months<br>6 months<br>9 months<br>12 months<br>3 years	Yes	No	Yes; SS	No	Yes	No	No	No

© 2025 Jarrar F et al. *JAMA Network Open.*

			5 years								
Yang, 2023[64]	Yes	First dialysis	3, 5, 8 years	Yes	Yes	Yes; SS	No	Yes	Yes; plot	Yes	Yes; nomogra m
Okada, 2024[65]	Yes	First dialysis	12 months	Yes	Yes	Yes; SS	Yes; temporal	Yes	No	No	No

Abbreviations: CV, cross-validation; BS, bootstrapping; SS, single split; HL, Hosmer-Lemeshow.

© 2025 Jarrar F et al. *JAMA Network Open.*

eTable 6. Characteristics of the Studies Included in the Systematic Review and Critical Appraisal for Risk of Bias and Applicability According to PROBAST

Author, Year	Modeling method	Sample size	Events n (%)	No predictors Cand.	No predictors Final	EPV or EPP	Selection of candidate predictors	Selection of final model	Number (%) and handling of missing data	Type of evaluation	Performance measures		Critical appraisal (PROBAST) P	Pr	O	A
Chen, 2014[16]	Cox regression	30,303	8537 (28.2)	13	13	656.7	Based on univariable associations	Stepwise and bootstrap; N#	n (%): Unknown Method: No information	Int: Bootstrap Ext : None	Cal: Calibration plot / HL test Disc : C-Statistic Ov: R-squared	RoB	-	-	+	-
												App	-	-	+	
Obi, 2018[17]	Cox regression	35,878	9813 (27.4)	46	34	213.3	No information	Backward elimination; Y#	n (%): 3600 (10.0) Method: Complete-case analysis	Int: Random split data Ext : Different setting	Cal: Calibration plot Disc : C-Statistic Ov: Not evaluated	RoB	-	+	+	-
												App	-	+	+	
Inaguma, 2019[18]	Cox regression	1,520	692 (45.5)	25	25	27.7	Based on prior knowledge	Unclear	n (%): Unknown Method: No information	Int: Bootstrap Ext : None	Cal: Not evaluated Disc : AUC graph / Log-rank test Ov: Not evaluated	RoB	-	?	-	-
												App	-	?	-	
Santos, 2020[19]	Logistic regression †	421	60 (14.3)	16	7	3.8	Based on univariable associations	No information	n (%): Unknown Method: No information	Int: Bootstrap Ext : None	Cal: HL test Disc : C-Statistic / AUC graph Ov: Not evaluated	RoB	?	+	+	-
												App	?	+	+	
Pladys, 2020[20]	Cox regression	9,052	1302 (14.4)	25	7	52.1	Based on univariable associations	No information	n (%): Unknown Method: Multiple imputation	Int: Random split data Ext : None	Cal: Calibration plot Disc : C-Statistic / AUC graph Ov: Not evaluated	RoB	+	+	?	-
												App	+	+	?	
Hemke, 2013[21]	Logistic regression †	13,868	8321 (60.0)	23	4	361.8	Based on univariable associations	Backward elimination; N#	n (%): 216 (1.6) Method: Multiple imputation	Int: Random split data Ext : None	Cal: Calibration plot Disc : C-Statistic Ov: Not evaluated	RoB	+	+	?	-
												App	?	+	?	
Chen, 2017[22]	Logistic regression	159,362	128199 (80.4)	35	22	3662.8	Based on univariable associations	No information	n (%): 8031 (5.0) Method: Complete-case analysis	Int: Random split data Ext : None	Cal: None Disc : C-Statistic / Log-rank test / Risk group curves Ov: Not evaluated	RoB	+	+	?	-
												App	+	+	+	
Chua, 2014[23]	Logistic regression	983	169 (17.2)	13	7	13.0	Based on univariable associations	Stepwise selection; N#	n (%): Unknown Method: No information	Int: None (Apparent performance) Ext : None	Cal: HL test / 0.73 (3mo), 0.21 (12mo) Disc : C-Statistic / Log-rank test / Risk group curves Ov: Not evaluated	RoB	-	-	-	-
												App	-	-	?	

© 2025 Jarrar F et al. *JAMA Network Open.*

van Dieppen, 2014[24]	Logistic regression †	394	82 (20.8)	14	7	5.9	Based on univariable associations	Backward elimination; Y#	n (%): Unknown Method: Multiple imputation	Int: Bootstrap Ext : None	Cal: Calibration plot / Slope Disc : C-Statistic / AUC graph Ov: Not evaluated	**RoB**	+	+	?	-
												**App**	+	?	?	
Floege, 2015[25]	Cox regression	9,722	1060 (10.9)	21	14	50.5	Based on univariable associations	Other	n (%): 500 (5.1) Method: Multiple imputation	Int: None (Apparent performance) Ext : Temporal	Cal: Calibration plot Disc : AUC graph / Risk group curves Ov: Not evaluated	**RoB**	+	?	+	-
												**App**	?	?	+	
Dusseaux, 2015[26]	Logistic regression	8,955	5104 (57.0)	20	14	192.6	Based on univariable associations	Unclear	n (%): Unknown Method: Multiple imputation	Int: None (Apparent performance) Ext : Temporal	Cal: Calibration plot / Slope / HL test / 0.2 Disc : C-Statistic Ov: Not evaluated	**RoB**	+	+	+	-
												**App**	-	+	+	
Doi, 2015[27]	Logistic regression	688	62 (9.0)	29	6	2.1	Based on univariable associations	Backward elimination; U#	n (%): 183 (26.6) Method: Multiple imputation	Int: Bootstrap Ext : None	Cal: Calibration plot Disc : C-Statistic / Risk group curves Ov: Not evaluated	**RoB**	-	-	?	-
												**App**	-	?	?	
Thamer, 2015[28]	Logistic regression	52,796	6477 (12.3)	32	7 (simple mod) ; 14 (complex mod)	202.4	Based on univariable associations and clinical relevance	Backward elimination; N#	n (%): 29237 (55.4) Method: Multiple imputation	Int: None (Apparent performance) Ext : Temporal	Cal: Calibration plot Disc : C-Statistic / Risk group curves Ov: Not evaluated	**RoB**	+	+	-	-
												**App**	?	+	?	
Couchoud, 2015[29]	Logistic regression	24,348	2548 (10.5)	15	9	169.9	Based on univariable associations	Other	n (%): Unknown Method: Multiple imputation	Int: Random split data Ext : None	Cal: Not evaluated Disc : C-Statistic / Risk group curves Ov: Not evaluated	**RoB**	+	+	+	-
												**App**	?	+	+	
Hemke, 2015[30]	Cox regression †	1,835	1101 (60.0)	23	10	31.9	Based on univariable associations and clinical relevance	Backward elimination; U#	n (%): 214 (11.7) Method: Multiple imputation	Int: Random split data Ext : None	Cal: Calibration plot / Slope Disc : C-Statistic Ov: Not evaluated	**RoB**	?	?	+	-
												**App**	?	-	+	
Patzer, 2016[31]	Logistic regression	663,860	265544 (40.0)	17	8	15620.2	Based on univariable associations	Backward elimination; N#	n (%): 89 (0.0) Method: Complete-case analysis	Int: Random split data Ext : None	Cal: Calibration plot Disc : C-Statistic Ov: Not evaluated	**RoB**	-	+	?	-
												**App**	-	?	?	
Haapio, 2017[32]	Logistic regression †	4,335	597 (13.8)	32	7	18.7	Based on univariable associations	Stepwise selection; N#	n (%): Unknown Method: Complete-case analysis	Int: None (Apparent performance) Ext : Temporal	Cal: Calibration plot / HL test Disc : C-Statistic Ov: Not evaluated	**RoB**	+	?	?	-
												**App**	+	?	?	

© 2025 Jarrar F et al. *JAMA Network Open.*

Lin, 2019[33]	RF and NN (binary)	48,153	10411 (21.6)	22	unclear	473.2	Unclear	Unclear	n (%): Unknown Method: No information	Int: Cross-validation Ext : None	Cal: Not evaluated Disc : C-Statistic Ov: Not evaluated	RoB	-	?	?	-
												App	-	?	?	
Akbilgic, 2019[34]	RF (binary)	27,615	6516 (23.6)	49	15	133.0	Other	Other	n (%): 8263 (29.9) Method: Complete-case analysis	Int: Cross-validation Ext : None	Cal: Not evaluated Disc : C-Statistic / AUC graph Ov: Not evaluated	RoB	-	+	?	-
												App	-	+	?	
Cho, 2017[35]	Cox regression	7,606	3003 (39.5)	17	unclear	176.6	All available predictors	Pre-specified model (not selection)	n (%): Unknown Method: No information	Int: None (Apparent performance) Ext : Temporal	Cal: Not evaluated Disc : C-Statistic / AUC graph / Log-rank test / Risk group curves Ov: Not evaluated	RoB	-	+	-	-
												App	-	?	+	
Wick, 2017[36]	Logistic regression	2,199	375 (17.1)	37	7	10.1	Based on univariable associations	Forward selection; U#	n (%): Unknown Method: No information	Int: Cross-validation Ext : None	Cal: Calibration plot / HL test Disc : C-Statistic / AUC graph Ov: Not evaluated	RoB	+	+	+	-
												App	?	+	+	
Ivory, 2017[37]	Logistic regression	23,658	1495 (6.3)	12	12	124.6	Based on univariable associations	Pre-specified model (not selection)	n (%): 111 (0.5) Method: Complete-case analysis	Int: None (Apparent performance) Ext : Temporal	Cal: Calibration plot / HL test Disc : C-Statistic Ov: Brier score	RoB	-	+	+	-
												App	-	+	+	
Geddes, 2006[38]	Cox regression	2,310	1217 (52.7)	54	24	20.2	All available predictors	Unclear	n (%): 78 (3.4) Method: Complete-case analysis	Int: None (Apparent performance) Ext : Temporal	Cal: Not evaluated Disc : AUC graph / Log-rank test Ov: Not evaluated	RoB	-	-	-	-
												App	-	-	-	
Mauri, 2008[39]	Logistic regression	5,738	946 (16.5)	10	10	94.6	All available predictors	Pre-specified model (not selection)	n (%): Unknown Method: No information	Int: Random split data Ext : None	Cal: HL test / P = 0.97 Disc : C-Statistic Ov: Not evaluated	RoB	?	+	?	-
												App	?	+	?	
Couchoud, 2009[40]	Logistic regression	4,991	470 (9.4)	19	9	24.7	Based on univariable associations	Bootstrap selection; N#	n (%): 849 (17.0) Method: Multiple imputation	Int: Random split data Ext : None	Cal: Calibration plot / HL test Disc : C-Statistic / Log-rank test / Risk group curves Ov: Not evaluated	RoB	+	+	+	-
												App	?	+	+	
Liu, 2010[41]	Cox regression	33,077	NA	15	15	Unknown	Based on univariable associations	Pre-specified model (not selection)	n (%): Unknown Method: No information	Int: None (Apparent performance) Ext : Temporal	Cal: Not evaluated Disc : C-Statistic / Log-rank test / Risk group curves Ov: Not evaluated	RoB	-	-	?	-
												App	-	-	?	
		242,576	NA	42		Unknown			n (%): Unknown		Cal: Not evaluated	RoB	-	-	?	-

© 2025 Jarrar F et al. *JAMA Network Open.*

Jacob, 2010[42]	Linear regression and NN (time, unclear cens.)				uncle ar		Based on univariable associations	Stepwise selection; N#	Method: Complete-case analysis	Int: Random split data Ext : None	Disc : Not evaluated Ov: Not evaluated	App	-	-	?	
Marinovich, 2010[43]	Cox regression	5,360	1091 (20.4)	19	19	57.4	All available predictors	Pre-specified model (not selection)	n (%): Unknown Method: Complete-case analysis	Int: None (Apparent performance) Ext : None	Cal: Not evaluated Disc : C-Statistic / AUC graph / Log-rank test / Risk group curves Ov: Not evaluated	RoB	-	+	-	-
												App	?	+	?	
Quinn, 2011[44]	Logistic regression †	16,205	1326 (8.2)	55	15	24.1	Based on univariable associations	Stepwise and bootstrap (unclear); U#	n (%): Unknown Method: No information	Int: Bootstrap Ext : None	Cal: Calibration plot / HL test Disc : C-Statistic Ov: Not evaluated	RoB	+	+	-	-
												App	+	+	?	
Wu, 2022[45]	Logistic regression	210,174	74574 (35.5)	11	11	6779.5	Based on univariable associations	Stepwise selection; N#	n (%): 1594 (0.8) Method: Complete-case analysis	Int: Cross-validation Ext : None	Cal: Calibration plot Disc : C-Statistic Ov: Not evaluated	RoB	-	+	-	-
												App	-	+	?	
Noh, 2020[46]	Logistic/Cox regression, RF, XGB and NN (bin./surv.)	1,730	343 (19.8)	72	23	4.8	Based on univariable associations	No information	n (%): Unknown Method: No information	Int: Unclear Ext : None	Cal: Not evaluated Disc : C-Statistic Ov: Not evaluated	RoB	-	?	+	-
												App	-	-	+	
Siddiqa, 2021[47]	Cox regression	758	481 (63.5)	18	5	15.4	Based on univariable associations	No information	n (%): Unknown Method: No information	Int: Random split data Ext : Geographical	Cal: Slope Disc : C-Statistic / D-Statistics / Risk group curves Ov: Not evaluated	RoB	+	-	+	-
												App	-	-	+	
McAdams-DeMarco, 2018[48]	Cox regression	1,975	207 (10.5)	16	15	12.9	Based on univariable associations	Pre-specified model (not selection)	n (%): Unknown Method: Complete-case analysis	Int: None (Apparent performance) Ext : None	Cal: Not evaluated Disc : C-Statistic Ov: Not evaluated	RoB	?	-	-	-
												App	-	-	-	
Gao, 2022[49]	Cox regression	200	45 (22.5)	9	5	5.0	Based on univariable associations	Backward elimination; N#	n (%): Unknown Method: No information	Int: None (Apparent performance) Ext : None	Cal: Not evaluated Disc : C-Statistic / AUC graph Ov: Not evaluated	RoB	-	-	-	-
												App	-	-	?	
Chaudhuri, 2023[50]	XGB (binary)	76,113	#VALUE!	23	23	Unknown	Based on prior knowledge	Stepwise selection; N#	n (%): Unknown Method: No information	Int: Random split data Ext : None	Cal: Not evaluated Disc : C-Statistic / AUC graph Ov: Not evaluated	RoB	-	?	?	-
												App	-	?	+	
Thijssen, 2012[51]	Logistic regression	4,512	258 (5.7)	20	4	12.9	No information	No information	n (%): Unknown Method: No information	Int: Random split data Ext : None	Cal: Not evaluated Disc : C-Statistic / AUC graph Ov: Not evaluated	RoB	?	-	?	-
												App	?	?	?	
	Cox regression	3,631		19		56.7		Unclear	n (%): Unknown		Cal: Calibration plot	RoB	-	-	+	-

© 2025 Jarrar F et al. *JAMA Network Open.*

Wagner, 2011[52]			1078 (29.7)		12 (20)		Based on prior knowledge		Method: Multiple imputation	Int: Random split data<br>Ext : None	Disc : C-Statistic<br>Ov: Not evaluated	App	-	-	+	
Zhu, 2021[53]	Cox regression	173	43 (24.9)	7	7	6.1	Based on univariable associations	Unclear	n (%): Unknown<br>Method: No information	Int: None (Apparent performance)<br>Ext : None	Cal: Calibration plot<br>Disc : C-Statistic / AUC graph<br>Ov: Brier score	RoB	?	-	?	-
												App	-	-	?	
Cohen, 2010[54]	Cox regression	512	123 (24.0)	9	5	13.7	Based on univariable associations	Stepwise selection; N#	n (%): 62 (12.1)<br>Method: Complete-case analysis	Int: None (Apparent performance)<br>Ext : Temporal	Cal: Not evaluated<br>Disc : C-Statistic / Log-rank test / Risk group curves<br>Ov: Not evaluated	RoB	-	-	?	-
												App	-	-	?	
Siga, 2020[55]	Logistic regression, Bayesian networks (binary)	4,915	1674 (34.1)	35	14	47.8	Unclear	Unclear	n (%): Unknown<br>Method: Complete-case analysis	Int: Cross-validation<br>Ext : None	Cal: Calibration plot<br>Disc : C-Statistic / AUC graph<br>Ov: Not evaluated	RoB	-	?	-	-
												App	-	?	-	
Jung, 2018[56]	Cox regression †	3,309	661 (20.0)	19	19	34.8	All available predictors	Pre-specified model (not selection)	n (%): Unknown<br>Method: No information	Int: Cross-validation<br>Ext : None	Cal: Calibration plot<br>Disc : C-Statistic / AUC graph<br>Ov: Not evaluated	RoB	-	+	+	-
												App	-	+	+	
Wang, 2021[57]	Neural network (binary)	1,200	145 (12.1)	64	64	2.3	All available predictors	Pre-specified model (not selection)	n (%): Unknown<br>Method: No information	Int: Cross-validation<br>Ext : None	Cal: Not evaluated<br>Disc : C-Statistic / AUC graph<br>Ov: Not evaluated	RoB	-	-	-	-
												App	-	-	-	
Tapak, 2020[58]	RF (survival)	785	376 (47.9)	28	28	13.4	All available predictors	Unclear	n (%): Unknown<br>Method: No information	Int: Bootstrap<br>Ext : None	Cal: Not evaluated<br>Disc : C-Statistic<br>Ov: Not evaluated	RoB	-	+	+	-
												App	-	+	+	
Holme, 2012[59]	Cox regression †	1,868	880 (47.1)	29	5	30.3	All available predictors	Stepwise selection; N#	n (%): Unknown<br>Method: No information	Int: Random split data<br>Ext : None	Cal: HL test<br>Disc : C-Statistic<br>Ov: Not evaluated	RoB	-	?	+	-
												App	-	-	+	
Rankin, 2022[60]	XGB (binary)	1,150,195	86083 (7.5)	188	unclear	457.9	All available predictors	Other	n (%): Unknown<br>Method: Multiple imputation	Int: Random split data<br>Ext : None	Cal: Calibration plot<br>Disc : C-Statistic / AUC graph<br>Ov: Not evaluated	RoB	?	?	+	-
												App	-	-	+	
Fernandez Lucas, 2007[61]	Cox regression	304	93 (30.6)	12	12	7.8	All available predictors	Pre-specified model (not selection)	n (%): Unknown<br>Method: No information	Int: None (Apparent performance)<br>Ext : None	Cal: Not evaluated<br>Disc : AUC graph / Log-rank test / Risk group curves<br>Ov: Not evaluated	RoB	-	+	+	-
												App	-	?	?	
		42,351	#VALUE!	44	44	Unknown	Unclear		n (%): Unknown			RoB	+	-	+	-

© 2025 Jarrar F et al. *JAMA Network Open.*

Goldstein, 2024[62]	LASSO & XGB (binary and survival)							LASSO selection; U#	Method: No information	Int: Random split data<br>Ext : None	Cal: Calibration plot / Slope / CITL<br>Disc : AUC graph<br>Ov: Not evaluated	**App**	+	-	+	
Noppakun, 2023[63]	Cox regression †	17,354	6309 (36.4)	24	7	262.9	Based on univariable associations	Backward elimination; U#	n (%): Unknown<br>Method: No information	Int: Random split data<br>Ext : None	Cal: Not evaluated<br>Disc : C-Statistic<br>Ov: Not evaluated	**RoB**	+	+	+	-
												**App**	+	+	+	
Yang, 2023[64]	Cox regression	551	207 (37.6)	11	7	18.8	No information	Pre-specified model (not selection)	n (%): Unknown<br>Method: No information	Int: Random split data<br>Ext : None	Cal: Calibration plot<br>Disc : AUC graph<br>Ov: Not evaluated	**RoB**	+	+	+	-
												**App**	+	+	+	
Okada, 2024[65]	Logistic regression (for score dev.)	2,739	424 (15.5)	16	9	26.5	No information	No information	n (%): Unknown<br>Method: Complete-case analysis	Int: Random split data<br>Ext : Temporal	Cal: Not evaluated<br>Disc : C-Statistic / AUC graph<br>Ov: Not evaluated	**RoB**	-	+	+	-
												**App**	-	+	+	

Abbreviations: Int, internal testing; Ext, external testing; Cal, calibration; CITL, calibration in the large; Disc, discrimination; Ov, overall; EPV, events per variable; EPP, events per parameter.
*These studies used machine learning techniques but methods for correct handling right censoring data were uncertain. † These studies reported regression coefficients and intercept or baseline survival information.
Accounted for optimism, yes, no or unclear (Y/N/U). XGB: extreme gradient boosting machines. RF: random forest algorithm. NN: neural networks.

© 2025 Jarrar F et al. *JAMA Network Open.*

eAppendix. Study Protocol

Last updated on September 30, 2024, date of the last search

This systematic review will be conducted according to the guidelines set out in the CHecklist for critical Appraisal and data extraction for systematic Reviews of prediction Modelling Studies (CHARMS)[1] and the Prediction model Risk Of Bias Assessment Tool (PROBAST).[2,3] The CHARMS checklist provides guidance for formulating the review question, and for extracting data from the primary studies reporting prediction models. The PROBAST tool includes four domains: participants, predictors, outcome, and analysis. For each domain the tool provides signaling questions for determining whether the risk of bias and the applicability should be graded as low, high or unclear. This systematic review will be reported according to the Transparent Reporting of multivariable prediction models for Individual Prognosis Or Diagnosis reporting guideline for systematic reviews of multivariable prediction models (TRIPOD-SRMA).[4] TRIPOD-SRMA contains 26 items and has superseded previous reporting guidelines.
We plan to conduct a systematic review without meta-analysis.

Systematic review registration
https://www.crd.york.ac.uk/prospero/display_record.php?ID=CRD42023486220
Registration date: December 5, 2023, last update October 10, 2024

Review question
What is the quality and clinical applicability of existing mortality prediction models for people with kidney failure?

Searches and sources
An information specialist and medical librarian (D.L.) will search Ovid MEDLINE, Ovid EMBASE, and the Cochrane Library from 2004, when the Kidney Disease: Improving Global Outcomes (KDIGO) was originally established to develop and implement guidelines for the care of people with kidney disease, to a date close to the submission date of the final manuscript. Searches will combine terms from three concepts: 1) chronic kidney failure (e.g., CKD, renal insufficiency); 2) mortality (e.g., mortality, death) and 3) prediction modelling (e.g., calibration, measures of discrimination) and recommended filters for prediction models.[3] Terms will be searched as keywords and subject headings (e.g., MEDLINE MeSH). No language restrictions will be applied to the search strategy. A complete description of the search strategy is included in PROSPERO. The reference list of all included articles will also be searched for any additional, relevant articles. Study citations and abstracts will be uploaded to Covidence to aid in study screening.

Study eligibility criteria
This review will include prospective and retrospective cohort studies that created or evaluated (with internal or external testing) mortality prediction models for people with kidney failure not yet treated with dialysis, choosing conservative care for kidney failure or commencing any form of dialysis (i.e., incident hemodialysis or peritoneal dialysis), with the outcome of all-cause mortality or cause-specific mortality and at least 3 months of prediction time horizon. Letters, editorials, narrative reviews, commentaries, and case reports will be excluded. Also, prediction models for people who received a kidney transplant will be excluded due to their improved prognosis after kidney transplantation.

© 2025 Jarrar F et al. *JAMA Network Open.*

Condition or domain being studied
Kidney failure, defined as eGFR of less than 15 ml/min/1.73 m^2 sustained for more than 90 days or requirement for kidney replacement with long-term dialysis (hemodialysis or peritoneal dialysis) or transplantation.

Participants/population
People with kidney failure who were or were not yet treated for end-stage kidney failure at the study entry date. Treatment included conservative care for kidney failure or dialysis. Transplant patients have better prognosis and thus studies including exclusively transplant patients will be excluded. Patients will be considered 'incident' dialysis patients if the study entry date (prediction time origin) was the dialysis initiation date. Patients will be considered 'prevalent' dialysis patients if they had been already on dialysis when they entered the study.

Main outcome
Mortality, from specific causes and all-cause mortality.

Data extraction
Two reviewers (F.J. and M.P.) will independently screen all abstracts of studies based on titles and abstracts, and then reviewed full texts in a second stage of the review to determine eligibility based on the study eligibility criteria described above.
Reviewers will use the CHARMS and the PROBAST checklists to extract data on study design, including study characteristics, prediction framework, model design, selection, training, and testing strategies, prediction performance measures, and applicability. Reviewers will also consider the extent to which each study adhered to the TRIPOD+AI reporting standard for all prediction models, irrespective of whether the primary study used regression or machine learning methods.[5] Discrepancies between the two reviewers in study selection for inclusion and data extraction will be resolved by discussing with an arbitrator (P.R.).
The following study characteristics will be pre-specified and extracted for each study (CHARMS items): author, publication year, country, setting, data source, study design, number of participants, study start year, duration of follow-up, inclusion criteria, exclusion criteria, dialysis modality (if any), incident vs. prevalent dialysis proportion, primary predictors and primary outcome. Reviewers will also independently extract all the elements of the study prediction framework; these will include target population, time origin, prediction time horizon, target of the analysis (individual risk of death), predictive variables, competing risks, model users and model evaluation (internal and external).

Risk of bias (quality) assessment
Reviewers will follow the PROBAST recommendations to evaluate the risk of bias and the applicability of included studies. Bias is the presence of systematic error in a study that may lead to distorted or flawed results hampering the study's internal validity. Prediction models are at high risk of bias when there are shortcomings in study design, conduct, or analysis that could lead to systematic bias in the predicted risks or in the estimates of a model's predictive performance. Applicability concerns of a model arise when the study that developed the model defined or captured data on population, predictors, or outcomes in ways that do not reflect the clinical setting where the model is intended to be used (target population, type and timing of measurement of the predictors and outcome).

Risk prediction framework
A medical risk prediction model should be developed in a framework that defines the target population, the prediction time origin (when the model is applied to predict the risk of a new patient), the

© 2025 Jarrar F et al. *JAMA Network Open.*

prediction time horizon, the predictors, the outcome, and competing events (if any). The framework implicitly defines who can use the model, how and when.[6,7]

Modelling algorithm and evaluation of prediction performance
There are many different ways to specify a regression model, and also many ways to tune a machine algorithm such as a random forest or a neural network. By modelling algorithm we mean all data dependent steps of modelling, including data dependent selection of predictor variables and data dependent tuning of hyperparameters, that are needed to produce the prediction model based on a dataset. For the resulting prediction model to be useful for new patients, in particular to avoid bias due to overfitting, it is crucial that the modelling algorithm is tested with some form of data-splitting. Cross-validation simulates the real-life situation where the model is trained using a dataset (training data) and then applied to inform new patients about the risk of the outcome. Most cross-validation approaches repeatedly split the data into non-overlapping training and test datasets. The procedure applies all steps of the modelling algorithm, including variable selection and tuning of hyperparameters, using the training dataset, and then evaluates the prediction performance in the test dataset.[7] For the review, reviewers will assess the modeling algorithms that the primary studies used to make the prediction model. Reviewers will consider modeling algorithms inappropriate if they based the selection of the predictors on univariate analyses and then used expert guided model building and goodness of fit testing to obtain the prediction model.[2,3] Reviewers will also consider backward variable selection inappropriate,[8] unless the modeling algorithm was evaluated using cross-validation where all steps of the modelling algorithm, including the backward selection, were repeated in training sets and evaluated in independent test sets.
Reviewers will assess whether the primary study used a single split of the data or cross-validation for model testing using the learning data (internal testing). A single random split is not recommended because the results will typically depend on the random seed (Monte-Carlo error), it is prone to manipulation and conceals part of the learning data.[7] Finally, reviewers will assess whether the final model was evaluated using data the model had not seen during learning (external testing based on temporally or geographically distinct data). Of note, we will intentionally avoid the use of the term 'validation' throughout our reporting,[9] as any statistical model falls short of the complexities of reality and thus cannot ultimately be considered valid. Instead, whether a prediction model is useful (or potentially harmful) should ultimately be tested in a randomized trial.[10]

Prediction models and key performance measures
Studies that follow people until death are characterized by right censored data. Reviewers will evaluate if authors had used appropriate statistical methods for right censored data and provided details on how censoring was handled (see PROBAST, Analysis domain, signaling question 6).
Reviewers will extract the criteria for model evaluation and comparison of rival models, including calibration plots and the time-dependent area under the receiver operating characteristic curve, AUC, a measure of discrimination, and the time-dependent Brier score or prediction error, a measure of both calibration and discrimination.[7] Reviewers will also report if the primary study had used improper performance measures, including the c-index (Harrell concordance index)[11] and measures of reclassification.[12,13] These measures are not proper because they may erroneously show that a mis-specified model systematically outperforms the data-generating model (the true model).[7]

Other criteria for model evaluation
Reviewers will assess whether study authors applied decision curve analysis,[14] and whether they linked specific clinical decisions with categories defined by the predicted risks.[15] Finally, reviewers will consider whether a model was tested in a clinical trial, as this is the ultimate test of model utility.[10] For

© 2025 Jarrar F et al. *JAMA Network Open.*

usability, reviewers will note whether authors provided a calculator, a nomogram, or an alternative tool that would ease the access to patient predicted risks.

© 2025 Jarrar F et al. *JAMA Network Open.*

eReferences.

1. Moons KGM, de Groot JAH, Bouwmeester W, et al. Critical Appraisal and Data Extraction for Systematic Reviews of Prediction Modelling Studies: The CHARMS Checklist. *PLoS Med*. 2014;11(10):e1001744. doi:10.1371/journal.pmed.1001744
2. Wolff RF, Moons KGM, Riley RD, et al. PROBAST: A Tool to Assess the Risk of Bias and Applicability of Prediction Model Studies. *Ann Intern Med*. 2019;170(1):51. doi:10.7326/M18-1376
3. Moons KGM, Wolff RF, Riley RD, et al. PROBAST: A Tool to Assess Risk of Bias and Applicability of Prediction Model Studies: Explanation and Elaboration. *Ann Intern Med*. 2019;170(1):W1. doi:10.7326/M18-1377
4. Snell KIE, Levis B, Damen JAA, et al. Transparent reporting of multivariable prediction models for individual prognosis or diagnosis: checklist for systematic reviews and meta-analyses (TRIPOD-SRMA). *BMJ*. Published online May 3, 2023:e073538. doi:10.1136/bmj-2022-073538
5. Collins GS, Moons KGM, Dhiman P, et al. TRIPOD+AI statement: updated guidance for reporting clinical prediction models that use regression or machine learning methods. *BMJ*. Published online April 16, 2024:e078378. doi:10.1136/bmj-2023-078378
6. Kattan MW, Gerds TA. A Framework for the Evaluation of Statistical Prediction Models. *Chest*. 2020;158(1S):S29-S38. doi:10.1016/j.chest.2020.03.005
7. Gerds Kattan M.W. TA. Medical risk prediction models: With ties to Machine Learning. Published online 2021. doi:10.1201/9781138384484
8. Austin PC, Tu J V. Automated variable selection methods for logistic regression produced unstable models for predicting acute myocardial infarction mortality. *J Clin Epidemiol*. 2004;57(11):1138-1146. doi:10.1016/j.jclinepi.2004.04.003
9. Van Calster B, Steyerberg EW, Wynants L, van Smeden M. There is no such thing as a validated prediction model. *BMC Med*. 2023;21(1):70. doi:10.1186/s12916-023-02779-w
10. Simon R. Clinical Trial Designs for Evaluating the Medical Utility of Prognostic and Predictive Biomarkers in Oncology. *Per Med*. 2010;7(1):33-47. doi:10.2217/pme.09.49
11. Blanche P, Kattan MW, Gerds TA. The c-index is not proper for the evaluation of t-year predicted risks. *Biostatistics*. 2019;20(2):347-357. doi:10.1093/biostatistics/kxy006
12. Pepe MS, Fan J, Feng Z, Gerds T, Hilden J. The Net Reclassification Index (NRI): a Misleading Measure of Prediction Improvement Even with Independent Test Data Sets. *Stat Biosci*. 2015;7(2):282-295. doi:10.1007/s12561-014-9118-0
13. Hilden J, Gerds TA. A note on the evaluation of novel biomarkers: do not rely on integrated discrimination improvement and net reclassification index. *Stat Med*. 2014;33(19):3405-3414. doi:10.1002/sim.5804
14. Vickers AJ, Elkin EB. Decision Curve Analysis: A Novel Method for Evaluating Prediction Models. *Medical Decision Making*. 2006;26(6):565-574. doi:10.1177/0272989X06295361
15. Liu P, Sawhney S, Heide-Jørgensen U, et al. Predicting the risks of kidney failure and death in adults with moderate to severe chronic kidney disease: multinational, longitudinal, population based, cohort study. *BMJ*. Published online April 15, 2024:e078063. doi:10.1136/bmj-2023-078063
16. Chen JY, Tsai SH, Chuang PH, et al. A Comorbidity Index for Mortality Prediction in Chinese Patients with ESRD Receiving Hemodialysis. *Clin J Am Soc Nephrol*. 2014;9(3):513-519. doi:10.2215/CJN.03100313
17. Obi Y, Nguyen DV, Zhou H, et al. Development and Validation of Prediction Scores for Early Mortality at Transition to Dialysis. *Mayo Clin Proc*. 2018;93(9):1224-1235. doi:10.1016/j.mayocp.2018.04.017
18. Inaguma D, Morii D, Kabata D, et al. Prediction model for cardiovascular events or all-cause mortality in incident dialysis patients. Shimosawa T, ed. *PLOS ONE*. 2019;14(8):e0221352. doi:10.1371/journal.pone.0221352

© 2025 Jarrar F et al. *JAMA Network Open.*

19. Santos J, Oliveira P, Malheiro J, et al. Predicting 6-Month Mortality in Incident Elderly Dialysis Patients: A Simple Prognostic Score. *Kidney Blood Press Res*. 2020;45(1):38-50. doi:10.1159/000504136
20. Pladys A, Vigneau C, Raffray M, et al. Contribution of medico-administrative data to the development of a comorbidity score to predict mortality in End-Stage Renal Disease patients. *Sci Rep*. 2020;10(1):8582. doi:10.1038/s41598-020-65612-x
21. Hemke AC, Heemskerk MB, Van Diepen M, Weimar W, Dekker FW, Hoitsma AJ. Survival prognosis after the start of a renal replacement therapy in the Netherlands: a retrospective cohort study. *BMC Nephrol*. 2013;14(1):258. doi:10.1186/1471-2369-14-258
22. Chen LX, Josephson MA, Hedeker D, Campbell KH, Stankus N, Saunders MR. A Clinical Prediction Score to Guide Referral of Elderly Dialysis Patients for Kidney Transplant Evaluation. *Kidney Int Rep*. 2017;2(4):645-653. doi:10.1016/j.ekir.2017.02.014
23. Chua HR, Lau T, Luo N, et al. Predicting First-Year Mortality in Incident Dialysis Patients with End-Stage Renal Disease - The UREA5 Study. *Blood Purif*. 2014;37(2):85-92. doi:10.1159/000357640
24. Van Diepen M, Schroijen MA, Dekkers OM, et al. Predicting Mortality in Patients with Diabetes Starting Dialysis. Moysés RMA, ed. *PLoS ONE*. 2014;9(3):e89744. doi:10.1371/journal.pone.0089744
25. Floege J, Gillespie IA, Kronenberg F, et al. Development and validation of a predictive mortality risk score from a European hemodialysis cohort. *Kidney Int*. 2015;87(5):996-1008. doi:10.1038/ki.2014.419
26. Dusseux E, Albano L, Fafin C, et al. A simple clinical tool to inform the decision-making process to refer elderly incident dialysis patients for kidney transplant evaluation. *Kidney Int*. 2015;88(1):121-129. doi:10.1038/ki.2015.25
27. Doi T, Yamamoto S, Morinaga T, Sada K ei, Kurita N, Onishi Y. Risk Score to Predict 1-Year Mortality after Haemodialysis Initiation in Patients with Stage 5 Chronic Kidney Disease under Predialysis Nephrology Care. Shimosawa T, ed. *PLOS ONE*. 2015;10(6):e0129180. doi:10.1371/journal.pone.0129180
28. Thamer M, Kaufman JS, Zhang Y, Zhang Q, Cotter DJ, Bang H. Predicting Early Death Among Elderly Dialysis Patients: Development and Validation of a Risk Score to Assist Shared Decision Making for Dialysis Initiation. *Am J Kidney Dis*. 2015;66(6):1024-1032. doi:10.1053/j.ajkd.2015.05.014
29. Couchoud CG, Beuscart JBR, Aldigier JC, Brunet PJ, Moranne OP. Development of a risk stratification algorithm to improve patient-centered care and decision making for incident elderly patients with end-stage renal disease. *Kidney Int*. 2015;88(5):1178-1186. doi:10.1038/ki.2015.245
30. Hemke AC, Heemskerk MBA, Van Diepen M, Dekker FW, Hoitsma AJ. Improved Mortality Prediction in Dialysis Patients Using Specific Clinical and Laboratory Data. *Am J Nephrol*. 2015;42(2):158-167. doi:10.1159/000439181
31. Patzer RE, Basu M, Larsen CP, et al. iChoose Kidney: A Clinical Decision Aid for Kidney Transplantation Versus Dialysis Treatment. *Transplantation*. 2016;100(3):630-639. doi:10.1097/TP.0000000000001019
32. Haapio M, Helve J, Grönhagen-Riska C, Finne P. One- and 2-Year Mortality Prediction for Patients Starting Chronic Dialysis. *Kidney Int Rep*. 2017;2(6):1176-1185. doi:10.1016/j.ekir.2017.06.019
33. Lin SY, Hsieh MH, Lin CL, et al. Artificial Intelligence Prediction Model for the Cost and Mortality of Renal Replacement Therapy in Aged and Super-Aged Populations in Taiwan. *J Clin Med*. 2019;8(7):995. doi:10.3390/jcm8070995
34. Akbilgic O, Obi Y, Potukuchi PK, et al. Machine Learning to Identify Dialysis Patients at High Death Risk. *Kidney Int Rep*. 2019;4(9):1219-1229. doi:10.1016/j.ekir.2019.06.009
35. Cho H, Kim MH, Kim HJ, et al. Development and Validation of the Modified Charlson Comorbidity Index in Incident Peritoneal Dialysis Patients: A National Population-Based Approach. *Perit Dial Int J Int Soc Perit Dial*. 2017;37(1):94-102. doi:10.3747/pdi.2015.00201

© 2025 Jarrar F et al. *JAMA Network Open.*

36. Wick JP, Turin TC, Faris PD, et al. A Clinical Risk Prediction Tool for 6-Month Mortality After Dialysis Initiation Among Older Adults. *Am J Kidney Dis*. 2017;69(5):568-575. doi:10.1053/j.ajkd.2016.08.035
37. Ivory SE, Polkinghorne KR, Khandakar Y, et al. Predicting 6-month mortality risk of patients commencing dialysis treatment for end-stage kidney disease. *Nephrol Dial Transplant*. Published online January 10, 2017:gfw383. doi:10.1093/ndt/gfw383
38. Geddes CC, Van Dijk PCW, McArthur S, et al. The ERA-EDTA cohort study—comparison of methods to predict survival on renal replacement therapy. *Nephrol Dial Transplant*. 2006;21(4):945-956. doi:10.1093/ndt/gfi326
39. Mauri JM, Cleries M, Vela E, Registry CR. Design and validation of a model to predict early mortality in haemodialysis patients. *Nephrol Dial Transplant*. 2008;23(5):1690-1696. doi:10.1093/ndt/gfm728
40. Couchoud C, Labeeuw M, Moranne O, et al. A clinical score to predict 6-month prognosis in elderly patients starting dialysis for end-stage renal disease. *Nephrol Dial Transplant*. 2009;24(5):1553-1561. doi:10.1093/ndt/gfn698
41. Liu J, Huang Z, Gilbertson DT, Foley RN, Collins AJ. An improved comorbidity index for outcome analyses among dialysis patients. *Kidney Int*. 2010;77(2):141-151. doi:10.1038/ki.2009.413
42. Jacob AN, Khuder S, Malhotra N, et al. Neural Network Analysis to Predict Mortality in End-Stage Renal Disease: Application to United States Renal Data System. *Nephron Clin Pract*. 2010;116(2):c148-c158. doi:10.1159/000315884
43. Marinovich S, Lavorato C, Moriñigo C, et al. A New Prognostic Index for One-Year Survival in Incident Hemodialysis Patients. *Int J Artif Organs*. 2010;33(10):689-699. doi:10.1177/039139881003301001
44. Quinn RR, Laupacis A, Hux JE, Oliver MJ, Austin PC. Predicting the Risk of 1-Year Mortality in Incident Dialysis Patients: Accounting for Case-Mix Severity in Studies Using Administrative Data. *Med Care*. 2011;49(3):257-266. doi:10.1097/MLR.0b013e318202aa0b
45. Wu MY, Hu PJ, Chen YW, et al. Predicting 3-month and 1-year mortality for patients initiating dialysis: a population-based cohort study. *J Nephrol*. 2022;35(3):1005-1013. doi:10.1007/s40620-021-01185-w
46. Noh J, Yoo KD, Bae W, et al. Prediction of the Mortality Risk in Peritoneal Dialysis Patients using Machine Learning Models: A Nation-wide Prospective Cohort in Korea. *Sci Rep*. 2020;10(1):7470. doi:10.1038/s41598-020-64184-0
47. Siddiqa M, Kimber AC, Shabbir J. Multivariable prognostic model for dialysis patients with end stage renal disease: An observational cohort study of Pakistan by external validation. *Saudi Med J*. 2021;42(7):714-720. doi:10.15537/smj.2021.42.7.20210082
48. McAdams-DeMarco MA, Ying H, Thomas AG, et al. Frailty, Inflammatory Markers, and Waitlist Mortality Among Patients With End-stage Renal Disease in a Prospective Cohort Study. *Transplantation*. 2018;102(10):1740-1746. doi:10.1097/TP.0000000000002213
49. Gao X, Wang J, Huang H, et al. Nomogram Model Based on Clinical Risk Factors and Heart Rate Variability for Predicting All-Cause Mortality in Stage 5 CKD Patients. *Front Genet*. 2022;13:872920. doi:10.3389/fgene.2022.872920
50. Chaudhuri S, Larkin J, Guedes M, et al. Predicting mortality risk in dialysis: Assessment of risk factors using traditional and advanced modeling techniques within the Monitoring Dialysis Outcomes initiative. *Hemodial Int*. 2023;27(1):62-73. doi:10.1111/hdi.13053
51. Thijssen S, Usvyat L, Kotanko P. Prediction of Mortality in the First Two Years of Hemodialysis: Results from a Validation Study. *Blood Purif*. 2012;33(1-3):165-170. doi:10.1159/000334138
52. Wagner M, Ansell D, Kent DM, et al. Predicting Mortality in Incident Dialysis Patients: An Analysis of the United Kingdom Renal Registry. *Am J Kidney Dis*. 2011;57(6):894-902. doi:10.1053/j.ajkd.2010.12.023

© 2025 Jarrar F et al. *JAMA Network Open.*

53. Zhu J, Tang C, Ouyang H, Shen H, You T, Hu J. Prediction of All-Cause Mortality Using an Echocardiography-Based Risk Score in Hemodialysis Patients. *Cardiorenal Med*. 2021;11(1):33-43. doi:10.1159/000507727
54. Cohen LM, Ruthazer R, Moss AH, Germain MJ. Predicting Six-Month Mortality for Patients Who Are on Maintenance Hemodialysis. *Clin J Am Soc Nephrol*. 2010;5(1):72-79. doi:10.2215/CJN.03860609
55. Siga MM, Ducher M, Florens N, et al. Prediction of all-cause mortality in haemodialysis patients using a Bayesian network. *Nephrol Dial Transplant*. 2020;35(8):1420-1425. doi:10.1093/ndt/gfz295
56. Jung HY, Kim SH, Jang HM, et al. Individualized prediction of mortality using multiple inflammatory markers in patients on dialysis. Wu PH, ed. *PLOS ONE*. 2018;13(3):e0193511. doi:10.1371/journal.pone.0193511
57. Wang Y, Zhu Y, Lou G, Zhang P, Chen J, Li J. A maintenance hemodialysis mortality prediction model based on anomaly detection using longitudinal hemodialysis data. *J Biomed Inform*. 2021;123:103930. doi:10.1016/j.jbi.2021.103930
58. Tapak L, Sheikh V, Jenabi E, Khazaei S. Predictors of mortality among hemodialysis patients in Hamadan Province using random survival forests. *J Prev Med Hyg*. Published online July 21, 2020:E482 Pages. doi:10.15167/2421-4248/JPMH2020.61.3.1421
59. Holme I, Fellström BC, Jardin AG, Schmieder RE, Zannad F, Holdaas H. Prognostic model for total mortality in patients with haemodialysis from the Assessments of Survival and Cardiovascular Events (AURORA) study. *J Intern Med*. 2012;271(5):463-471. doi:10.1111/j.1365-2796.2011.02435.x
60. Rankin S, Han L, Scherzer R, et al. A Machine Learning Model for Predicting Mortality within 90 Days of Dialysis Initiation. *Kidney360*. 2022;3(9):1556-1565. doi:10.34067/KID.0007012021
61. Fernandez Lucas M, Teruel JL, Zamora J, Lopez Mateos M, Rivera M, Ortuno J. A Mediterranean age-comorbidity prognostic index for survival in dialysis populations. *J Nephrol*. 2007;20(6):696-702.
62. Goldstein BA, Xu C, Wilson J, et al. Designing an Implementable Clinical Prediction Model for Near-Term Mortality and Long-Term Survival in Patients on Maintenance Hemodialysis. *Am J Kidney Dis*. 2024;84(1):73-82. doi:10.1053/j.ajkd.2023.12.013
63. Noppakun K, Nochaiwong S, Tantraworasin A, et al. Mortality Rates and a Clinical Predictive Model for the Elderly on Maintenance Hemodialysis: A Large Observational Cohort Study of 17,354 Asian Patients. *Am J Nephrol*. 2024;55(2):136-145. doi:10.1159/000535669
64. Yang M, Yang Y, Xu Y, et al. Development and Validation of Prediction Models for All-Cause Mortality and Cardiovascular Mortality in Patients on Hemodialysis: A Retrospective Cohort Study in China. *Clin Interv Aging*. 2023;Volume 18:1175-1190. doi:10.2147/CIA.S416421
65. Okada H, Ono A, Tomori K, et al. Development of a prognostic risk score to predict early mortality in incident elderly Japanese hemodialysis patients. McGrowder DA, ed. *PLOS ONE*. 2024;19(4):e0302101. doi:10.1371/journal.pone.0302101
