# Supplementary material for: Mortality Risk Prediction Models for People With Kidney Failure: A Systematic Review
Source: JAMA Netw Open. 2025 Jan 3;8(1):e2453190. doi: 10.1001/jamanetworkopen.2024.53190 (PMC11699530; doi:10.1001/jamanetworkopen.2024.53190)
Supplement: Supplement 2. — Data Sharing Statement [file jamanetwopen-e2453190-s002.pdf]

Data Sharing Statement

Jarrar. Mortality Risk Prediction Models for People With Kidney Failure. *JAMA Netw Open*. Published January 03, 2025. doi:10.1001/jamanetworkopen.2024.53190

Data
Data available: No

Additional Information
Explanation for why data not available: Data are publicly available, as this is a systematic review
